# Localized Delivery of the mRNAs Encoding CD47 Inhibitor and Interleukins 12, 15, and 21 Elicits Robust Antitumor Immunity

**DOI:** 10.1002/advs.202417205

**Published:** 2025-07-13

**Authors:** Tao Jiang, Shuaiyang Jing, Haojun Li, Bao Xiao, Jiahui Jin, Xiu Sun, Juan Wang, Jing Liang, Tongze Cai, Huili Hu, Meilan Wei, Xuanrui Zhong, Yang Ji, Peng George Wang, Jianlong Zhou

**Affiliations:** ^1^ Guangxi International Zhuang Medicine Hospital Guangxi University of Chinese Medicine Nanning 530200 China; ^2^ School of Medicine Southern University of Science and Technology Shenzhen 518055 China; ^3^ Key University Laboratory of Metabolism and Health of Guangdong Southern University of Science and Technology Shenzhen 518055 China; ^4^ Medi‐X Pingshan Southern University of Science and Technology Shenzhen 518055 China; ^5^ Shenzhen Maternity & Child Healthcare Hospital Shenzhen 518017 China

**Keywords:** CD47 inhibitor, cytokine, immunotherapy, lipid nanoparticle, mRNA therapeutics

## Abstract

CD47 inhibitors have emerged as promising candidates in cancer immunotherapy by activating macrophage phagocytosis through CD47/SIRPα signal blockade and enhancing dendritic cell antigen presentation. However, their clinical efficacy remains limited and is accompanied by severe side effects, including anemia and hemagglutination. To address these limitations, a localized delivery strategy is developed using lipid nanoparticles to encapsulate mRNA encoding a secreted CD47 inhibitor. This mRNA‐derived CD47 inhibitor effectively activated macrophages against cancer cells in vitro. To improve its modest tumor‐suppressive effects in vivo, a combination therapy is formulated by co‐delivering mRNAs encoding the CD47 inhibitor and a cytokine cocktail (interleukins 12, 15, and 21), achieving synergistic antitumor effects. Notably, this strategy induces robust systemic antitumor immunity and long‐lasting immunological memory, effectively suppressing metastatic tumor progression following localized treatment of the primary tumor. Mechanistically, the mRNA‐derived CD47 inhibitor promoted the differentiation of conventional type 1 dendritic cells, facilitating T cell cross‐priming and activating cytotoxic T cells. Additionally, the cytokine cocktail further augmented the antitumor activity of these cytotoxic T cells. These findings present a promising strategy for advancing clinical cancer treatment, addressing key challenges associated with current CD47 inhibitor therapies.

## Graphical abstract

The co‐delivery of mRNAs encoding a CD47 inhibitor and a cytokine cocktail (interleukins 12, 15, and 21) via lipid nanoparticles enables effective localized immunotherapy. This strategy not only suppresses tumor progression but also enhances type 1 conventional dendritic cell (cDC1) differentiation and activates CD8^+^ T cells, thereby inducing robust systemic antitumor immunity and establishing long‐lasting immunological memory to prevent tumor recurrence.

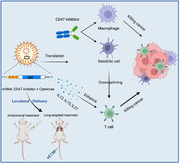

## 1 Introduction

Tumor microenvironment (TME) in solid tumors is a complex and dynamic system composed of various cell types, molecules and signaling pathways that interact with tumor cells to support tumor progression and immune evasion.^[^ ^1^ ^]^ The antitumor activities of immune cells within the TME are profoundly inhibited by many factors, including immunosuppressive molecules, hypoxia, acidic pH and nutrient deprivation.^[^ ^2^ ^]^ Reversing this immunosuppressive TME by enhancing T cell‐mediated antitumor functions has emerged as a promising strategy for cancer therapy.^[^ ^3^ ^]^ Approaches such as immune checkpoint blockade (ICB) therapies and adoptive T cell therapies showed strong potential in reinvigorating T cell cytotoxicity against tumors.^[^ ^4^ ^]^ However, the clinical efficacy of ICBs, such as programmed cell death protein 1 (PD‐1) and cytotoxic T‐lymphocyte associated protein 4 (CTLA‐4) inhibitors, was limited to a small proportion of patients.^[^ ^5^ ^]^ Similarly, while chimeric antigen receptor (CAR) T cell therapy has revolutionized the treatment of hematological malignancies, its effectiveness in solid tumors still remains a major challenge.^[^ ^6^ ^]^

Signal regulatory protein alpha (SIRPα) is an immune checkpoint molecule that plays a critical role in regulating macrophage phagocytosis.^[^ ^7^ ^]^ Upon binding to its ligand CD47, the two immunoreceptor tyrosine‐based inhibition motifs (ITIMs) of SIRPα in the cytoplasm undergo phosphorylation, recruiting Src homology region 2 domain‐containing phosphatase‐1/2 (SHP1/2) molecules.^[^ ^8^ ^]^ This cascade results in the dephosphorylation of myosin IIA, thereby inhibiting macrophage phagocytosis.^[^ ^8b^ ^]^ Under physiological conditions, aged red blood cells are cleared by macrophages due to the downregulation of CD47.^[^ ^9^ ^]^ Similarly, hematopoietic cells lacking CD47 were rapidly eliminated by macrophages after engraftment.^[^ ^10^ ^]^ However, many cancers evade immunological surveillance by overexpressing CD47.^[^ ^11^ ^]^ To target this mechanism, a variety of CD47 inhibitors have been developed for cancer therapy, including Hu5F9‐G4, TTI‐621, ALX‐148, TJC4 and IMM01.^[^ ^12^ ^]^ These agents consist of monoclonal antibodies targeting CD47 and SIRPα‐Fc fusion proteins. Previous studies suggested that blocking the CD47/SIRPα signaling axis with these CD47 inhibitors reactivated macrophages and effectively suppressed tumor progression in preclinical models.^[^ ^13^ ^]^ Moreover, CD47 blockade has been shown to promote dendritic cells (DCs) maturation and enhance T cell priming through activation of the cyclic GMP‐AMP synthase / stimulator of interferon genes (cGAS/STING) signaling pathway.^[^ ^14^ ^]^ Despite these promising findings, the clinical translation of CD47 inhibitors has been hindered by significant challenges. One major obstacle is their binding to CD47 on red blood cells, leading to dose‐limiting “on‐target, off‐tumor” toxicities such as anemia, hemagglutination, fever, and headache.^[^ ^15^ ^]^ Additionally, the therapeutic efficacy observed in clinical trials was relatively modest, further limiting their broad clinical utility.^[^ ^16^ ^]^

The combined strategy of simultaneously activating both innate and adaptive immune responses through CD47 inhibitors in conjunction with CAR‐T cell therapy or PD‐1/PD‐L1 inhibitors showed potent antitumor effects in several studies.^[^ ^17^ ^]^ However, ALX Oncology recently reported that the CD47 inhibitor Evorpacept in combination with the PD‐1 inhibitor pembrolizumab for patients with advanced head and neck squamous cell carcinoma (HNSCC) failed to meet their primary endpoints in phase II clinical trials (NCT04675294, NCT04675333). A possible explanation for this limited synergy is that PD‐1 inhibitors predominantly act on exhausted T cells within the TME, while the T cells activated by CD47 inhibition may not be responsive to PD‐1 blockade. Cytokines are key regulators of T cell‐mediated antitumor immunity, with some promoting tumor progression while others exert potent antitumor effects.^[^ ^18^ ^]^ Interleukins 12, 15, and 21 (IL‐12, IL15, and IL‐21) demonstrated positive antitumor activity in preclinical and clinical studies by supporting T cells activation, proliferation and persistence.^[^ ^19^ ^]^ Whether these interleukins can further potentiate the antitumor functions of the cytotoxic T cells activated by CD47 inhibitors remains to be elucidated. Notably, systemic administration of these cytokines was associated with dose‐dependent toxicities and a high risk of inducing cytokine release syndrome (CRS).^[^ ^20^ ^]^ In contrast, localized delivery may offer a safer and more focused strategy to enhance immune responses while minimizing systemic side effects.

Lipid nanoparticles (LNPs) are versatile tools widely employed for in vivo RNA delivery.^[^ ^21^ ^]^ This RNA‐based LNP technology has been utilized in various applications, including mRNA vaccines,^[^ ^22^ ^]^ protein replacement therapies,^[^ ^23^ ^]^ and CRISPR‐based gene editing therapeutics.^[^ ^24^ ^]^ LNP formulations typically comprise ionizable or cationic lipids, cholesterol, helper lipids, and poly(ethylene glycol) (PEG)ylated lipids.^[^ ^21^ ^]^ Traditional LNPs are known for their strong liver‐targeting properties in vivo. However, by incorporating specific cationic lipids into the formulation at optimized ratios, these LNPs were redirected to target organs such as the lungs or spleen.^[^ ^25^ ^]^ This enables precise delivery of mRNA therapeutics to diseased organs, enhancing their efficacy in disease treatment. For example, a previous study demonstrated that optimized lung‐targeted LNPs restored CFTR gene function in lung stem cells of cystic fibrosis mice through adenine base editing.^[^ ^26^ ^]^ Such organ‐targeted LNPs hold great potential for reducing the “on‐target, off‐tumor” toxicity of CD47 inhibitors in cancer therapy. Additionally, the targeted delivery of cytokines using these LNPs can further enhance antitumor response while minimizing systemic side effects.

In this study, we developed novel mRNA‐based therapeutics delivered by LNP, encoding a secreted CD47 inhibitor and a cytokine cocktail (IL‐12, IL‐15, and IL‐21) for localized cancer therapy. Our findings demonstrated that the combination of the CD47 inhibitor and the cytokine cocktail significantly potentiated antitumor activities with minimal toxic side effects across both immunologically “hot” and “cold” tumor types. Importantly, this combination therapy also induced robust systemic antitumor immunity and long‐term antitumor immune memory, leading to effective suppression of metastatic tumors following localized treatment of the primary tumor. Mechanistically, the mRNA‐derived CD47 inhibitor promoted the differentiation of type 1 conventional dendritic cells (cDC1s), thereby enhancing T cell priming. Concurrently, the cytokine cocktail synergistically activated and amplified the antitumor functions of cytotoxic T cells. In addition, this therapeutic strategy demonstrated comparable efficacy when delivered via lung‐targeted LNP in lung‐metastatic tumor models and achieved superior therapeutic outcomes compared to the combination of CD47 inhibitor mRNA and anti‐PD‐1 antibody. These findings offer a promising strategy to overcome the limitations of current CD47 inhibitor therapies and advance the development of mRNA‐based cancer immunotherapy.

## 2 Results

### 2.1 mRNA‐Encoded CD47 Inhibitor Activated Macrophage Phagocytosis In Vitro

**Figure 1:**
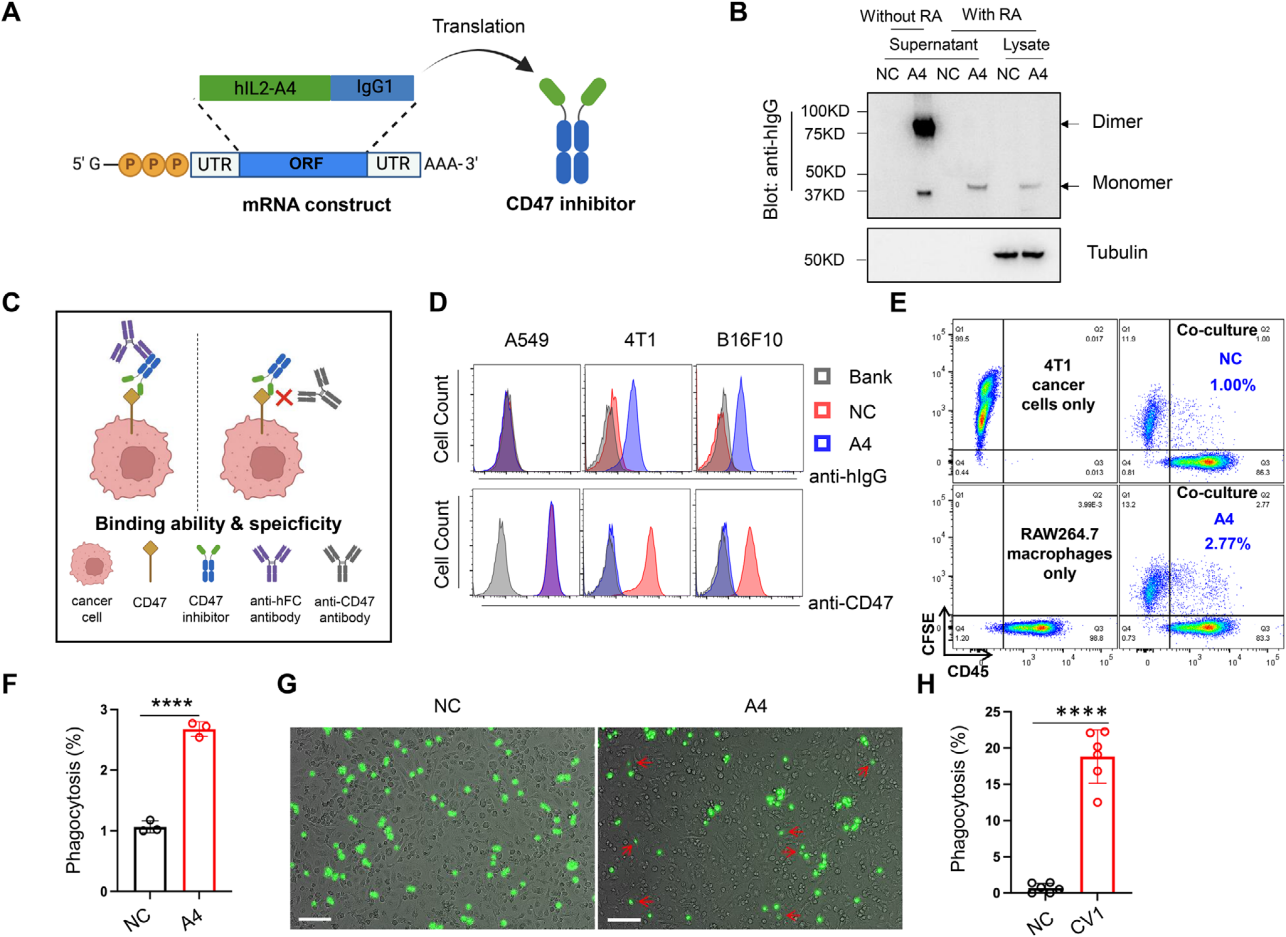
mRNA‐encoded CD47 inhibitor activated macrophage phagocytosis in vitro. A) Schematic illustration of the mRNA construct encoding A4‐IgG1. hIL2: human IL2 signal peptide; A4: A4 nanobody; IgG1: human IgG1 Fc. B) Western blot analysis of the mRNA‐encoded A4‐IgG1 fusion protein in cell culture supernatants and cell lysates of 293T cells 48 h post‐transfection. Protein samples were treated with or without the reducing agent (RA) before loading. NC: negative control; A4: A4 mRNA. C) Schematic illustration of the experiment design for assessing the binding ability and specificity of the mRNA‐encoded A4‐IgG1. D) Flow cytometry analysis of the binding ability (upper panel) and specificity (lower panel) of the mRNA‐encoded A4‐IgG1 in A549, 4T1, and B16F10 cell lines. E) The representative image of flow cytometry analysis of RAW264.7 macrophage phagocytosis against 4T1 cells 2 h after co‐culture. 4T1 cells were blocked by the mRNA‐derived A4‐IgG1 and labeled with CFSE dye before coculture. RAW264.7 cells were stained with anti‐mouse CD45 antibody after coculture. The effector‐to‐target (E:T) ratio was 5:1. This experiment was performed independently three times. F) Bar chart quantifying RAW264.7 macrophage phagocytosis induced by the mRNA‐encoded CD47 inhibitor. n=3. G) The representative image of primary macrophages phagocytosis against 4T1 cells following CD47 blockade by the mRNA‐derived A4‐IgG1. 4T1 cells were blocked by the mRNA‐derived A4‐IgG1 and labeled with CFSE dye before coculture. Red arrows indicated engulfed cells. The effector‐to‐target (E:T) ratio was 10:1. Six independent experiments were conducted. Scale bar: 300 µm. H) Bar chart quantifying primary macrophage phagocytosis from six independent experiments. n=6. In all panels, significance was determined by unpaired two‐tailed t‐test. Mean ± SD. ****P<0.0001. The illustrations were created with BioRender.com.

CD47 antibody is one of the widely used types of CD47 inhibitors designed to block CD47/SIRPα signaling.^[^ ^12^ ^]^ Based on the mouse CD47 nanobody A4,^[^ ^27^ ^]^ we designed a mRNA construct that contained a secretion signal at the 5′ end and a human IgG1 Fc domain at the 3′ end (**Figure** **1**A). Upon delivery into cells, this mRNA construct produced CD47 inhibitor proteins that were secreted and activated macrophages, mediating antibody‐dependent cellular phagocytosis through IgG1‐FcγR signaling.^[^ ^28^ ^]^ To confirm the translation and secretion of the mRNA, we synthesized the mRNA product via in vitro transcription (Figure S1A, Supporting Information) and transfected it into 293T cells. The supernatant was subsequently collected for protein detection. Western blot analysis revealed that the mRNA‐encoded A4‐IgG1 protein was successfully translated and secreted into the supernatant (Figure 1B).

Next, we screened mouse cancer cell lines to identify those with high CD47 expression (Figure S1B, Supporting Information). To evaluate the binding abilities and specificity of the mRNA‐derived CD47 inhibitor, cancer cells (A549, 4T1, and B16F10) were incubated with the supernatant containing the CD47 inhibitor. Following incubation, the cells were stained with either an anti‐human Fc antibody or an anti‐CD47 antibody and analyzed by flow cytometry (Figure 1C). The results demonstrated that the mRNA‐derived A4‐IgG1 specifically bound to mouse CD47 on the cancer cells (Figure 1D). To further assess the biological function of the mRNA‐derived CD47 inhibitor, the phagocytosis assay was performed by co‐culturing macrophages with cancer cells pre‐incubated with the supernatant. The results showed that cancer cells treated with the supernatant were significantly engulfed by macrophages compared to those without CD47 blockade (Figure 1E–H; Figure S1C, Supporting Information).

Therefore, these results demonstrate that the mRNA‐encoded CD47 inhibitor effectively enhances macrophage phagocytosis in vitro.

### 2.2 mRNA‐Encoded CD47 Inhibitor Promoted cDC1 Differentiation

**Figure 2:**
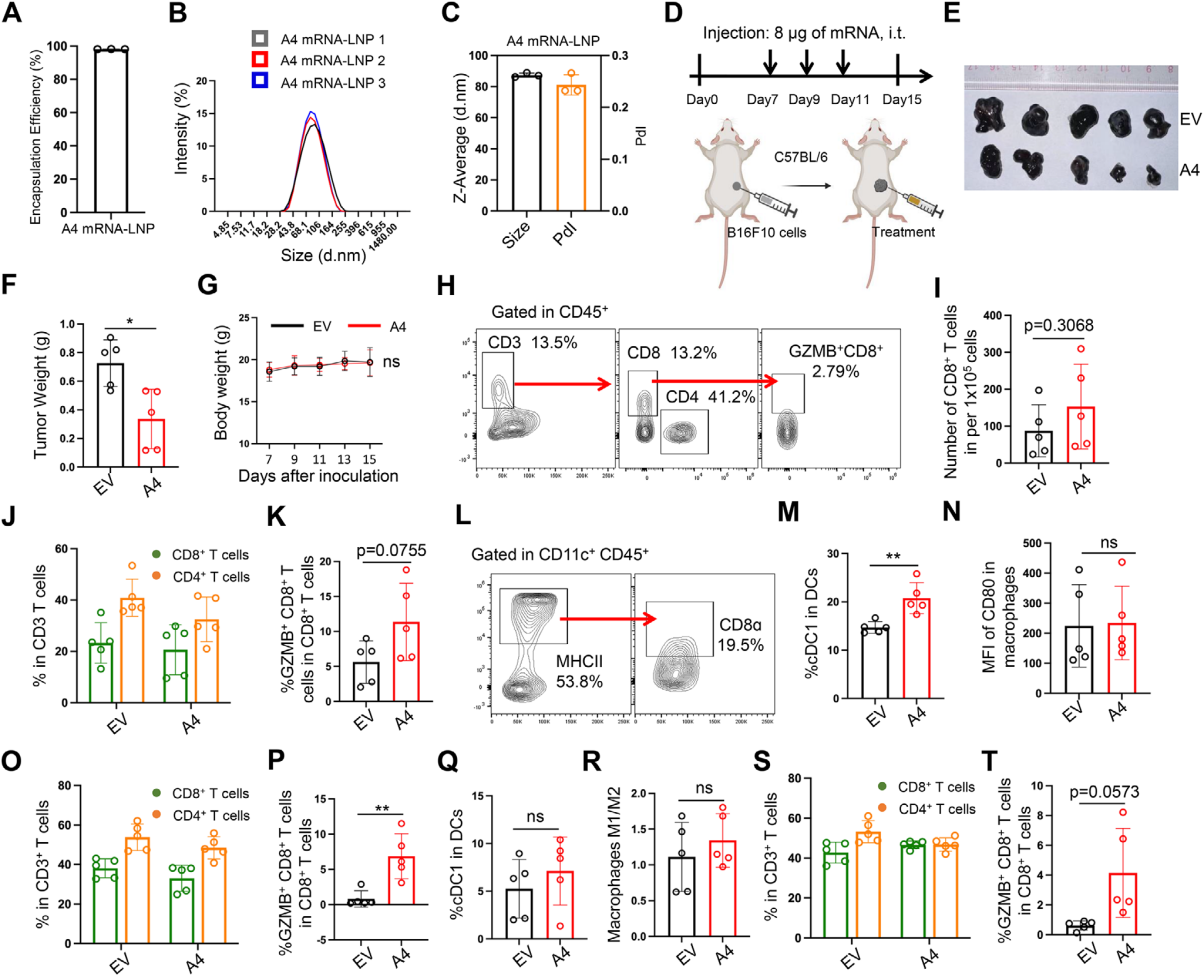
mRNA‐encoded CD47 Inhibitor Promoted cDC1 Differentiation. A‐C) The characterization of A4 mRNA encapsulated by DLin‐MC3 LNP. The encapsulation efficiency (A), particle size (B,C), and polydispersity index (PdI) (C) were shown. n=3. D) Schematic of the therapeutic regimen for subcutaneous melanoma models. i.t.: intratumoral injection. E) The images of tumors after treatments. EV: the mice treated with empty vehicle; A4: the mice treated with A4 mRNA. F) The bar chart quantifying the weight of melanoma. n=5. G) The body weight changes of the mice with treatments. n=5. H) Flow cytometry gating strategy for T cells analysis. I) Bar chart quantifying the number of infiltrating CD8^+^ T cells. n=5. J) The proportion of CD8^+^ T cells and CD4^+^ T cells in CD3^+^ T cells of tumors. n=5. K) Bar chart quantifying the percentage of GZMB^+^ CD8^+^ T cells in CD8^+^ T cells of tumors. n=5. L) The gating strategy of flow cytometry for cDC1 analysis. M) The percentage of cDC1 in DCs of tumors. n=5. N) The expression level of CD80 on macrophages of tumors. n=5. O,P) Flow cytometry analysis of T cells in splenocytes. The proportion of CD8^+^ T cells and CD4^+^ T cells in CD3^+^ T cells (O), and GZMB^+^ CD8^+^ T cells percentage in CD8^+^ T cells (P). n=5. Significance in panel P was determined by Mann Whitney test. Mean ± SD. Q,R) Flow cytometry analysis of cDC1 (Q) and macrophages (R) in splenocytes. n=5. S,T) Flow cytometry analysis of T cells in peripheral blood. The proportion of CD8^+^ T cells and CD4^+^ T cells in CD3^+^ T cells (S), and GZMB^+^ CD8^+^ T cells percentage in CD8^+^ T cells (T). n=5. Significance in panel T was determined by Mann Whitney test. Mean ± SD. In all panels, significance was determined by unpaired two‐tailed t‐test unless otherwise specified. Mean ± SD. *P < 0.05, **P<0.01. The illustrations were created with BioRender.com.

DLin‐MC3, a FDA‐approved lipid, has been widely used as a versatile vehicle for mRNA delivery in vivo. Using this formulation, A4‐IgG1 mRNA was efficiently encapsulated with high encapsulation efficiency (**Figure** **2**A). Both the particle size and polydispersity index (PdI) of the mRNA‐loaded nanoparticles were within normal ranges (Figure 2B,C). To evaluate the therapeutic efficacy of the mRNA‐encoded CD47 inhibitor in vivo, we established subcutaneous tumors by inoculating B16F10 melanoma cells into C57BL/6 mice, followed by intratumoral injection of 8 µg of mRNA (Figure 2D). After three doses of the mRNA‐LNP treatment, tumor growth was significantly suppressed compared to the control group (Figure 2E,F; Figure S2A, Supporting Information). To assess the safety profile of the treatment, complete blood counts were conducted. The results showed no significant changes in red blood cell, white blood cell, lymphocyte, or platelet counts (Figure S2B–E, Supporting Information). Furthermore, body weight remained stable in mice treated with A4 mRNA, comparable to that of control animals (Figure 2G). These findings suggest that localized delivery of mRNA‐encoded CD47 inhibitor effectively suppressed melanoma growth without inducing anemia. However, A4 mRNA showed only mild antitumor efficacy in 4T1 breast cancer and LLC1 lung cancer models—both classified as immunologically “cold” tumors (Figure S2F–I, Supporting Information).

To further explore immune activation, tumor‐infiltrating immune cells were analyzed following enzymatic digestion of melanoma tissue. Although A4 mRNA‐treated tumors exhibited higher numbers of CD8^+^ T cells, the increase was not statistically significant (Figure 2H,I). While the overall percentages of CD8^+^ and CD4^+^ T cells were comparable between two groups, the proportion of GZMB^+^ CD8^+^ T cells within the CD8^+^ population was higher in the A4 mRNA‐treated group (Figure 2J,K). In addition, A4 mRNA treatment significantly promoted the differentiation of cDC1s, which are essential for CD8^+^ T cells priming, whereas M1 macrophage polarization remained largely unchanged (Figure 2L–N). Consistent with intratumoral findings, splenocytes and peripheral blood from A4 mRNA‐treated mice also exhibited an increased proportion of GZMB^+^ CD8^+^ T cells (Figure 2P,T), despite no change in the overall ratios of CD8^+^ and CD4^+^ T cells (Figure 2O,S). The numbers of cDC1 cells and M1 macrophages in splenocytes were not significantly affected by A4 mRNA treatment (Figure 2Q,R). In contrast, A4 mRNA failed to increase the infiltration of activated CD8^+^ T cells in 4T1 breast tumors (Figure S2J, Supporting Information).

Collectively, these results indicate that locally delivered A4 mRNA can modulate the immune landscape in “hot” tumors like melanoma, primarily by promoting cDC1 differentiation and CD8^+^ T cell activation, thereby contributing to tumor suppression. However, this effect was not observed in “cold” tumors such as 4T1. Although the mRNA‐based CD47 inhibitor exhibited modest antitumor efficacy, its favorable safety profile underscores its potential as a safer therapeutic approach.

### 2.3 Combination of CD47 Inhibitor and A Cytokine Cocktail Synergistically Suppressed Tumor Progression

**Figure 3:**
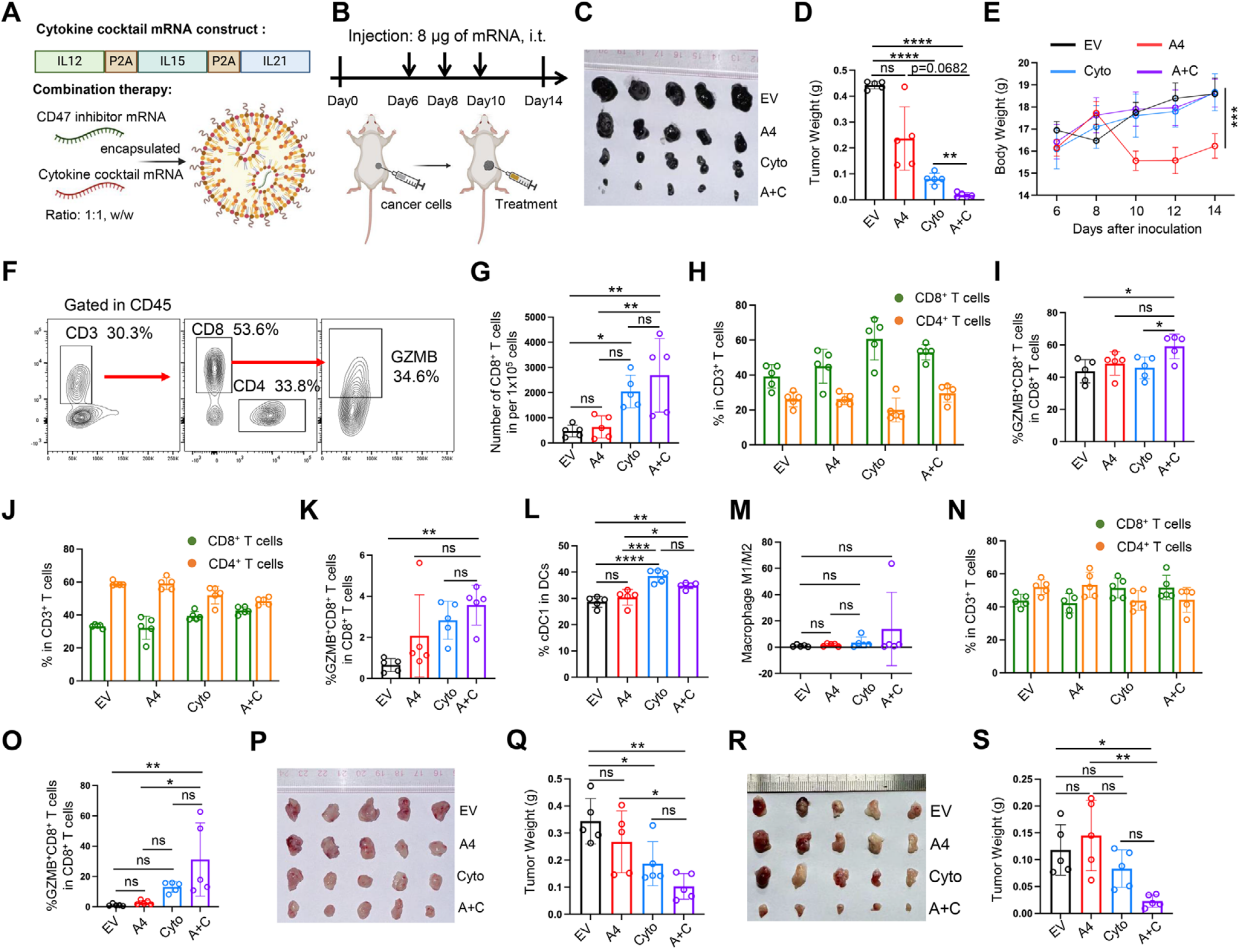
Combination of CD47 inhibitor and a cytokine cocktail synergistically suppressed tumor progression. A) Schematic illustration of the combination of A4 mRNA and the cytokine cocktail mRNA. B) Schematic of the combination therapy regimen for subcutaneous melanoma models. C) The images of tumors after treatments. EV: empty vehicle; A4: A4 mRNA; Cyto: cytokine cocktail mRNA; A+C: combination of A4 mRNA and cytokine cocktail mRNA. D) Bar chart quantifying the tumor weight after treatments. Significance was determined by Dunnett's T3 multiple comparisons test, mean ± SD. E) The body weight changes of the mice with treatments. F) Flow cytometry gating strategy for T cells analysis. G) Bar chart quantifying the number of infiltrating CD8^+^ T cells in tumor. H,I) Flow cytometry analysis of T cells in tumors. The proportion of CD8^+^ T cells and CD4^+^ T cells in CD3^+^ T cells (H), and GZMB^+^ CD8^+^ T cells percentage in CD8^+^ T cells (I). J,K) Flow cytometry analysis of T cells in splenocytes. The proportion of CD8^+^ T cells and CD4^+^ T cells in CD3^+^ T cells (J), and GZMB^+^ CD8^+^ T cells percentage in CD8^+^ T cells (K). L,M) Flow cytometry analysis of cDC1 (L) and macrophages (M) in splenocytes. N,O) Flow cytometry analysis of T cells in peripheral blood. The proportion of CD8^+^ T cells and CD4^+^ T cells in CD3^+^ T cells (N), and GZMB^+^ CD8^+^ T cells percentage in CD8^+^ T cells (O). P,Q) The efficacy of the combination therapy in 4T1 breast cancer. The images of tumors (P) and the tumor weight after treatments (Q). R,S) The efficacy of the combination therapy in LLC1 lung cancer. The images of tumors (R) and the tumor weight after treatments (S). In all panels, significance was determined by one‐way ANOVA with Tukey's multiple comparisons tests unless otherwise specified. *P<0.05, **P<0.01, ***P<0.001, ****P<0.0001, n=5, ns: not significant, mean ± SD. The illustrations were created with BioRender.com.

Previous studies have demonstrated that DCs, rather than macrophages, are primarily responsible for T cell priming and activation following CD47 antibody treatment.^[^ ^14^, ^29^ ^]^ Consistently, our findings revealed enhanced cDC1 differentiation and CD8^+^ T cell activation after A4 mRNA‐LNP administration (Figure 2M). However, the therapeutic effect of the CD47 inhibitor alone was modest (Figure 2E,F; Figure S2F–I, Supporting Information). Based on these observations, we hypothesized that combining a CD47 inhibitor with agents that stimulate and sustain T cell activation could produce a more potent antitumor response. IL‐12, IL‐15, and IL‐21 are well‐characterized for their pivotal roles in T cell activation, proliferation, and differentiation.^[^ ^19b^ ^]^ To test our hypothesis, we designed an mRNA construct encoding murine IL‐12, IL‐15, and IL‐21 simultaneously (**Figure** **3**A). This cytokine cocktail mRNA was mixed with A4 mRNA at a 1:1 w/w ratio and encapsulated in DLin‐MC3‐based LNPs for localized delivery (Figure 3A; Figure S3A, Supporting Information). In subcutaneous melanoma models, the mRNA mixture significantly suppressed tumor growth compared to treatment with either empty vehicle or the cytokine cocktail mRNA alone (Figure 3B–D; Figure S3B, Supporting Information). Given the known risk of CRS associated with cytokine‐based immunotherapies,^[^ ^20a^ ^]^ we evaluated the safety of the mRNA mixture. Neither body weight changes nor blood test results indicated signs of CRS or anemia following treatment (Figure 3E; Figure S3C–F, Supporting Information). However, those mice treated with A4 mRNA alone exhibited significant weight loss, despite having comparable red blood cell counts to the control group (Figure 3E; Figure S3C, Supporting Information). This isolated observation may have resulted from experimental variation, as it was not observed in prior experiments (Figure 2G). Importantly, the mRNA mixture also significantly inhibited tumor growth in both 4T1 breast cancer and LLC1 lung cancer models (Figure 3P–S), suggesting the synergistic antitumor efficacy of this combination strategy in both “hot” and “cold” tumors.

To assess the immune modulation within TME, we analyzed tumor‐infiltrating immune cells in melanoma. Both the mRNA mixture and the cytokine cocktail mRNA markedly increased intratumoral CD8^+^ T cell infiltration compared to the control (Figure 3F,G). While the total percentage of CD8^+^ T cells within CD3^+^ cells remained unchanged (Figure 3H), the proportion of GZMB^+^ CD8^+^ T cells was significantly elevated following the mRNA mixture treatment, indicating enhanced cytotoxic function (Figure 3I). Similar trends were observed in splenocytes and peripheral blood (Figure 3J,K,N,O). Furthermore, the mRNA mixture significantly enhanced cDC1 differentiation in splenocytes (Figure 3L), while showing no substantial effect on M1 macrophage polarization (Figure 3M). These results suggested that the mRNA combination therapy not only promoted CD8^+^ T cell activation in tumors but also increased the systemic pool of activated cytotoxic T cells. In addition, the mRNA mixture significantly elevated the infiltration of IFN‐γ^+^ CD8^+^ T cells in 4T1 tumors (Figure S3G, Supporting Information), further supporting its ability to reshape the immunosuppressive TME in cold tumors. The modest tumor‐suppressive effect observed with the cytokine cocktail monotherapy may be attributed to its ability to partially enhance CD8^+^ T cells infiltration within the tumors.

In conclusion, this study demonstrates that co‐delivery of CD47 inhibitor mRNA and a cytokine cocktail mRNA effectively reprograms the TME by promoting cDC1 differentiation and activating CD8^+^ T cells, leading to robust antitumor immunity.

### 2.4 The Combination Therapy Induced Systemic Antitumor Immunity and Long‐Lasting Immunological Memory

**Figure 4:**
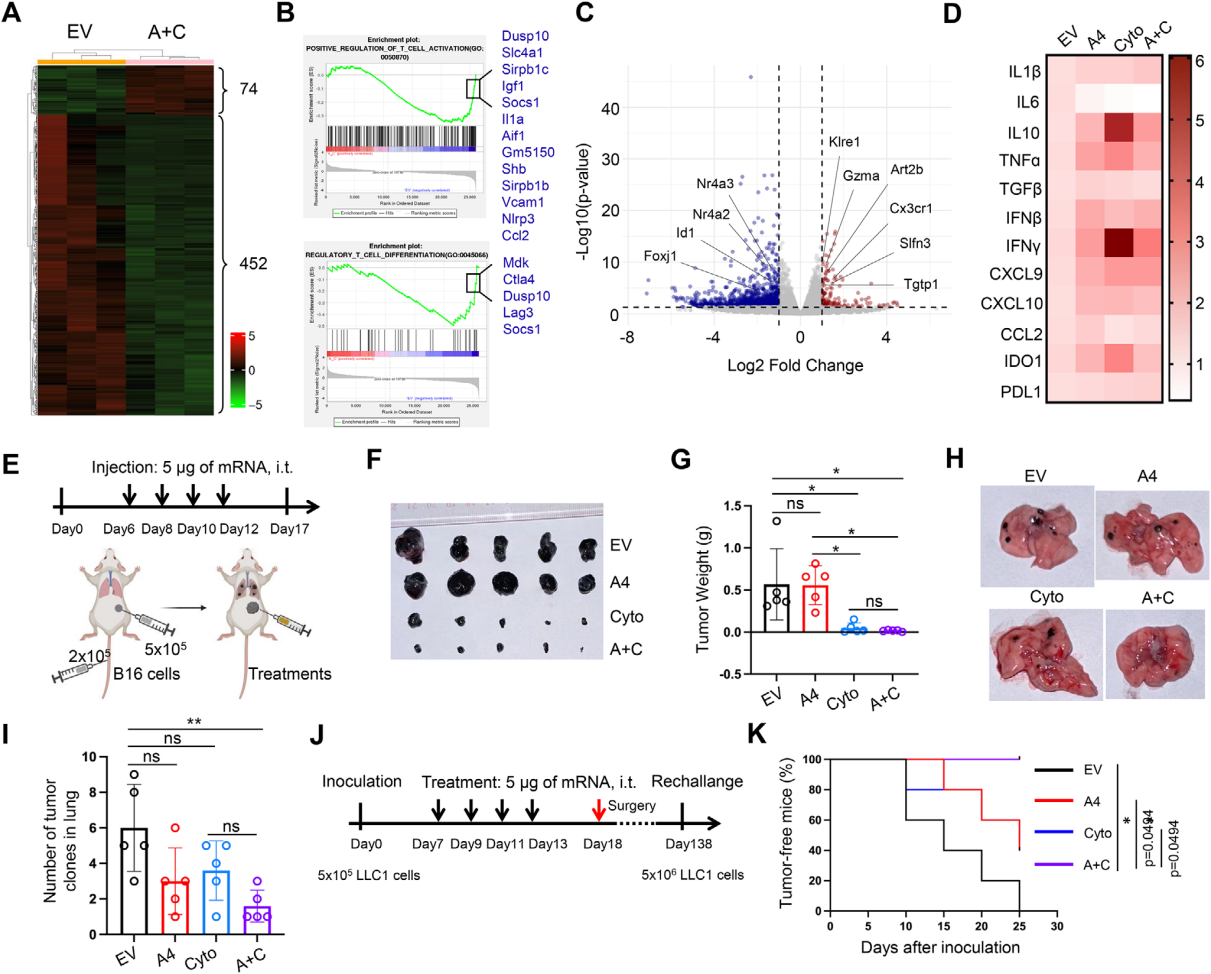
The combination therapy induced systemic antitumor immunity and long‐lasting antitumor memory. A) Cluster analysis of differentially expressed genes of the CD45^+^ CD3^+^ T cells isolated from splenocytes of BALB/c mice bearing 4T1 tumors. Compared to the control group, 74 genes were upregulated, and 452 genes were downregulated. n=3. B) GSEA analysis of differentially expressed genes, with representative core enrichment genes listed on the right. C) The volcano plot of differentially expressed genes. Grey dots represent non‐significant genes, red dots represent upregulated genes, and blue dots represent downregulated genes. D) Relative mRNA levels of different cytokines in 4T1 tumors growing in BALB/c mice. n=4. E) Schematic of the combination therapy regimen for melanoma models. F,G) The images of subcutaneous tumors after treatments (F), and the weights of subcutaneous tumors were quantified (G). n=5. H,I) The representative images of tumors in lung following the primary tumor treatments (H), and the number of tumor clones in lungs (I) were quantified. n=5. J) The design of tumor rechallenge experiment. K) Tumor‐free mice after rechallenge were quantified. n=5. Significance was determined by Log‐rank (Mantel‐Cox) test. In all panels, significance was determined by one‐way ANOVA with Tukey's multiple comparisons tests unless otherwise specified. *P < 0.05, **P < 0.01, ns: not significant, mean ± SD. The illustrations were created with BioRender.com.

To investigate the mechanism underlying the antitumor effects of the mRNA mixture, splenocytes were isolated from the treated BALB/c mice bearing 4T1 tumors. CD45^+^ CD3^+^ T cells were sorted for transcriptome analysis. Compared to the control group, 526 differentially expressed genes were identified in the mRNA mixture‐treated group, including 74 upregulated and 452 downregulated genes (**Figure** **4**A). Gene set enrichment analysis (GSEA) identified two phenotypes associated with T cell activation (Figure 4B). Among the core enrichment genes, Dusp10, Socs1, Gm5150, Shb, Vcam1, Nlrp3, Ctla4, and Lag3 were found to be involved in T cell immunosuppression and exhaustion (Figure 4B). Notably, several upregulated genes, including Gzma, Cx3cr1, Klre1, Art2b, Slfn3, and Tgtp1, were identified as key contributors to T cell‐mediated antitumor activity (Figure 4C). In contrast, transcription factors involved in regulating Treg cell differentiation and T cell exhaustion, such as Foxj1, Id1, Nr4a2, and Nr4a3, were significantly downregulated in the mRNA mixture‐treated group (Figure 4C). Therefore, these results suggested that T cells in the mRNA mixture‐treated mice exhibited a more activated, less exhausted phenotype. In addition, we further analyzed the expression of key inflammatory cytokines in 4T1 tumors. Tumors treated with the mRNA mixture displayed elevated expression of antitumor cytokines TNF‐α, IFN‐β, IFN‐γ, and CXCL9, while the level of the pro‐tumor cytokine IL‐6 was significantly decreased (Figure 4D). Other cytokines, including IL‐1β, TGF‐β, CXCL10, and CCL2, exhibited only modest changes (Figure 4D). Moreover, the inhibitory molecules, such as IDO1 and PD‐L1, showed no significant changes in their expression levels (Figure 4D).

Given that tumor metastasis is a leading cause of cancer‐related mortality and presents a major therapeutic challenge,^[^ ^30^ ^]^ we sought to determine whether the mRNA mixture provides therapeutic benefit in advanced‐stage disease. To this end, we established a mouse melanoma (B16F10) model in C57BL/6 mice bearing both subcutaneous and lung‐metastatic tumors simultaneously (Figure 4E), recapitulating the clinical scenario of melanoma with lung metastasis. Local administration of the mRNA mixture to the subcutaneous tumors led to significant inhibition of tumor growth, even at relatively low doses (Figure 4F,G). Notably, the progression of lung metastases was also markedly suppressed (Figure 4H,I), indicating that local delivery of the mRNA mixture induced a potent systemic antitumor response. To evaluate whether this combination therapy could establish long‐term antitumor memory, LLC1 tumors were surgically removed following treatment, and the tumor‐free C57BL/6 mice were rechallenged with the same LLC1 cells two months later (Figure 4J). Remarkably, none of the mice treated with the mRNA mixture developed recurrent tumors within 25 days of re‐inoculation. In contrast, all mice treated with empty vehicle relapsed, while only two mice in either the A4 mRNA or cytokine cocktail monotherapy group remained tumor‐free (Figure 4K). These findings suggested that the mRNA mixture not only promoted strong tumor clearance but also induced durable immunological memory.

In summary, these results demonstrate that the combination of CD47 inhibitor and the cytokine cocktail elicits a robust systemic antitumor response and long‐lasting antitumor immunity, effectively suppressing metastatic tumor progression and preventing recurrence.

### 2.5 The Combination Therapy Suppressed Lung‐Metastatic Tumor Progression via Targeted Delivery

**Figure 5:**
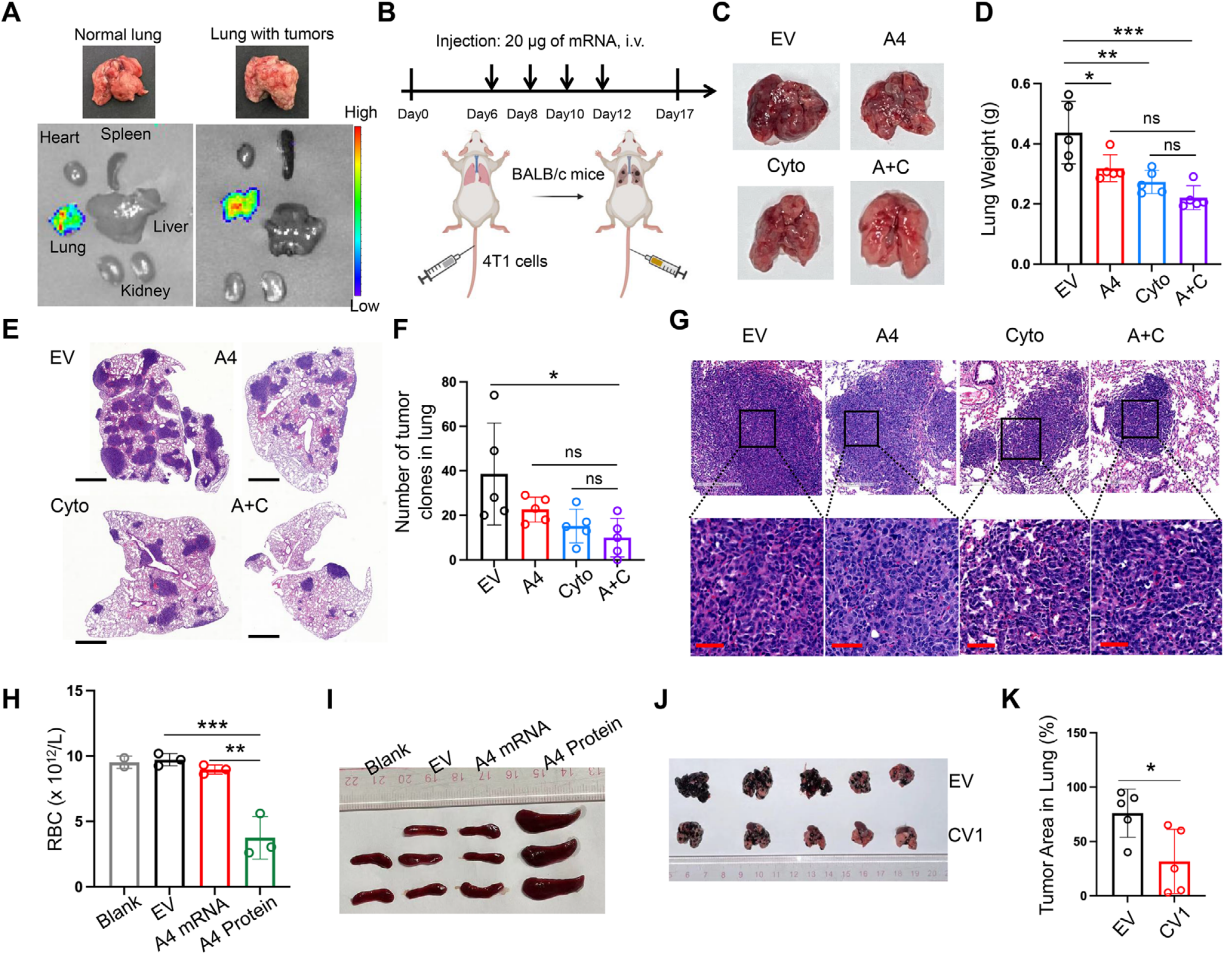
The combination therapy suppressed lung‐metastatic tumor progression via targeted delivery. A) Bioluminescent imaging of major organs from healthy mice and the mice bearing lung‐metastatic tumors after intravenous injection with 5 µg of firefly luciferase mRNA encapsulated DOTAP‐modified DLin‐MC3 LNP. Organs were collected for imaging 6 h post‐treatment. B) Schematic of the therapeutic regimen for lung‐metastatic tumor models. i.v.: intraveneous injection. C,D) The images of lungs (C) were shown and the weights of lungs were quantified (D). n=5. Significance was determined by one‐way ANOVA with Tukey's multiple comparisons tests. Mean ± SD. E‐G) The representative images of hematoxylin and eosin staining of tumors in lungs (E,G). The number of tumor clones in lungs were quantified in bar chart (F). Significance was determined by one‐way ANOVA with Tukey's multiple comparisons tests. Mean ± SD, n=5. H) Bar chart quantifying the number of red blood cells of the mice treated with empty vehicle, A4 mRNA and A4‐IgG1 protein. n=3. Significance was determined by one‐way ANOVA with Tukey's multiple comparisons tests. Mean ± SD. I) The images of spleen of the mice with treatments. J,K) The images of lungs with tumors were shown (J), and the tumor area in lungs was quantified after treatments (K). n=5. Significance was determined by unpaired two‐tailed t‐test. Mean ± SD. *P < 0.05, **P < 0.01, ***P < 0.001, ns: not significant. The illustrations were created with BioRender.com.

In recent years, the development of novel LNPs has enabled more precise and efficient targeted delivery of mRNA therapeutics.^[^ ^31^ ^]^ To evaluate the targeting specificity of the DOTAP‐modified DLin‐MC3 formulation, we encapsulated firefly luciferase mRNA into this LNP and administered it in vivo. The modified LNP demonstrated high mRNA encapsulation efficiency (Figure S4A, Supporting Information), with both particle size and polydispersity index (PdI) falling within normal ranges (Figure S4B,C, Supporting Information). Bioluminescent imaging revealed that this mRNA‐loaded LNP specifically targeted the lungs following intravenous injection, in both healthy mice and those bearing lung‐metastatic tumors (**Figure** **5**A; Figure S4D, Supporting Information). To assess the therapeutic potential of this lung‐targeted system for cancer treatment, we established lung‐metastatic tumor models in BALB/c mice using 4T1 cells, followed by intravenous administration of the mRNA mixture encapsulated in the DOTAP‐modified LNP (Figure 5B). Consistent with previous findings, this targeted delivery approach significantly inhibited lung‐metastatic tumor growth (Figure 5C–F). Histological analysis via hematoxylin and eosin staining further confirmed enhanced immune cell infiltration and reduced tumor malignancy grade in the lungs of treated mice (Figure 5G). These results demonstrated the potent antitumor efficacy of lung‐targeted delivery of the mRNA mixture.

To evaluate potential systemic side effects of this approach, peripheral blood was collected from healthy BALB/c mice treated with A4 mRNA. For comparison, purified A4‐IgG1 protein was administered intravenously at a dose of 2.5 mg/kg in a separate cohort (Figure S4E,F, Supporting Information). Hematological analysis showed that A4 mRNA treatment had negligible impact on red blood cell counts, whereas A4‐IgG1 protein administration led to a substantial reduction in red blood cells and induced splenomegaly (Figure 5H,I). Both A4 mRNA and A4‐IgG1 protein treatments did not significantly alter the levels of white blood cells, lymphocytes, and platelets (Figure S4G–I, Supporting Information). In tumor‐bearing mice, the mRNA mixture delivered via modified LNP had minimal effects on the counts of red blood cells, white blood cells, lymphocytes, monocytes, and platelets compared to the vehicle control (Figure S5A–E, Supporting Information). Furthermore, histopathological examination of major organs in healthy mice, including the lungs, liver, kidneys, spleen and heart, revealed no signs of organ toxicity following lung‐targeted administration with A4 mRNA, the cytokine cocktail mRNA or the mRNA mixture (Figure S5F, Supporting Information).

To further validate the antitumor efficacy of this lung‐targeted strategy, we applied the same approach using an alternative CD47 inhibitor, a mutant human SIRPα (CV1), in a NOD SCID mouse model bearing lung‐metastatic melanoma (Figure S5G, Supporting Information). Similar therapeutic benefits were observed (Figure 5J,K).

Together, these findings demonstrate that lung‐targeted delivery of CD47 inhibitor mRNA in combination with the cytokine cocktail mRNA represents a promising strategy for the treatment of lung cancers, offering effective tumor suppression with minimal systemic toxicity.

### 2.6 The Combination Therapy Exhibited Promising Clinical Translation Potential

**Figure 6:**
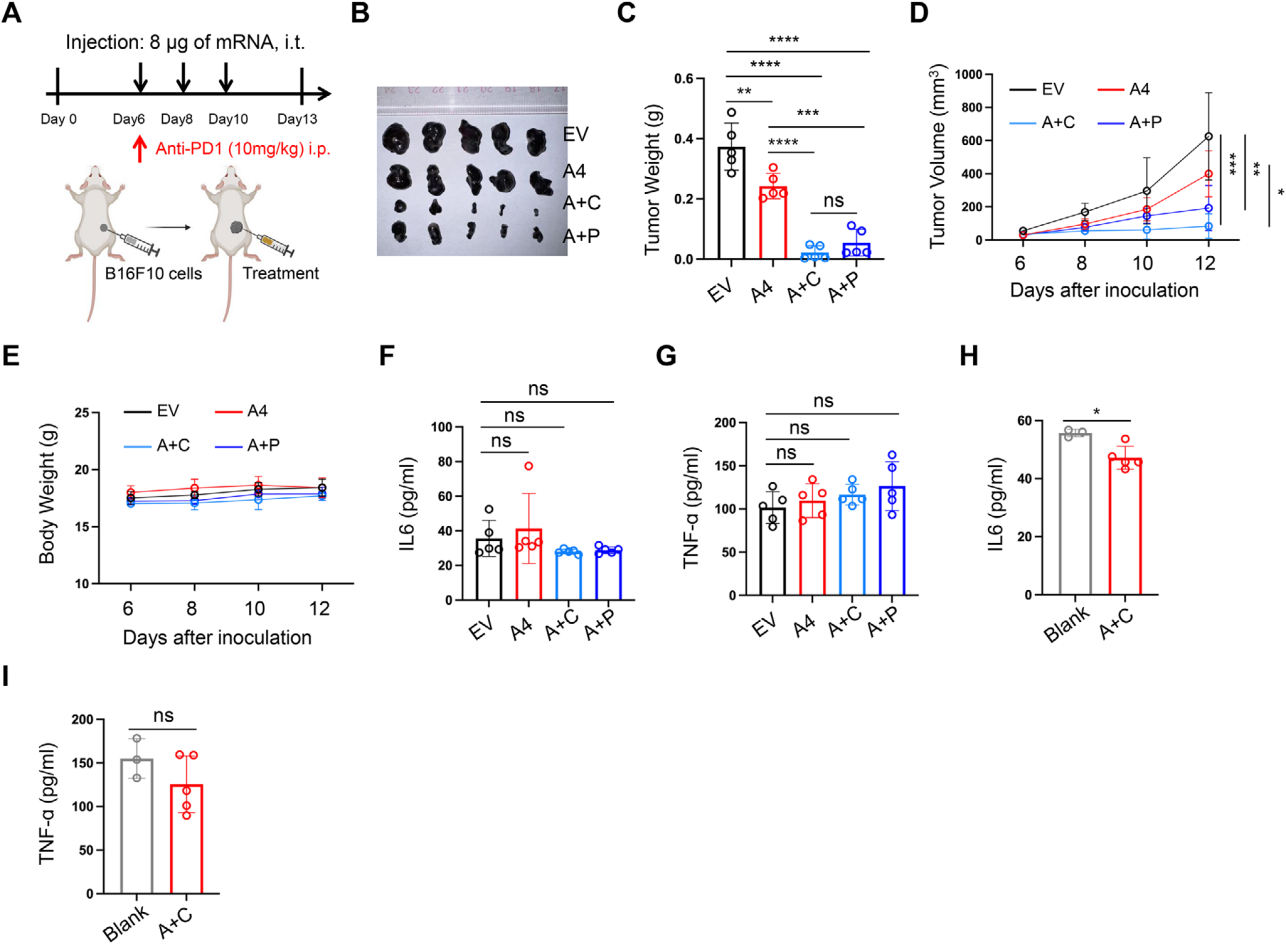
The combination therapy exhibited promising clinical translation potential. A) Schematic of the treatment regimen in subcutaneous melanoma models. i.t.: intratumoral injection. i.p.: intraperitoneal injection. B) Representative images of subcutaneous melanoma tumors following treatment. C) Quantification of tumor weights at the end of the experiment. A+P: combination of A4 mRNA and anti‐PD‐1 antibody. D) Tumor growth curves showing melanoma volumes over time. E) Body weight changes of treated mice over time. F,G) Serum levels of IL‐6 (F) and TNF‐α (G) in the short‐term treatment mice, measured by ELISA. H,I) Serum levels of H) IL‐6 and I) TNF‐α in the tumor‐free mice that survived for over eight months after tumor rechallenge, measured by ELISA. n(blank)=3. Significance was determined by unpaired two‐tailed t‐test. In all panels, significance was determined by one‐way ANOVA with Tukey's multiple comparisons tests unless otherwise specified. *P<0.05, **P<0.01, ***P<0.001, ****P<0.0001, n=5, ns: not significant, mean ± SD. The illustrations were created with BioRender.com.

Clinical trials evaluating the CD47 inhibitor Evorpacept in combination with the PD‐1 inhibitor pembrolizumab for patients with advanced HNSCC failed to meet their primary endpoints. To enhance the clinical relevance of our strategy, we conducted a comparative experiment in which the mRNA‐encoded CD47 inhibitor was combined with an anti‐PD‐1 antibody (**Figure** **6**A). While this combination significantly suppressed melanoma progression compared to the CD47 inhibitor alone, co‐delivery of the mRNA‐encoded CD47 inhibitor and cytokine cocktail demonstrated superior therapeutic efficacy (Figure 6B–D). None of the treatments caused notable body weight loss or altered the counts of red blood cells, white blood cells, lymphocytes, or platelets (Figure 6E; Figure S6A–D, Supporting Information). Moreover, serum levels of the inflammatory cytokines IL‐6 and TNF‐α remained comparable across treatment groups (Figure 6F,G), suggesting minimal risk of anemia or CRS.

To further evaluate long‐term safety, peripheral blood samples were collected from the tumor‐free mice that received the mRNA combination therapy and survived for over eight months in the tumor rechallenge experiment. Hematological parameters, including red blood cells, white blood cells, lymphocytes, and platelets, remained within normal ranges (Figure S6E–H, Supporting Information). TNF‐α levels were similar to those in untreated mice, and IL‐6 levels were significantly lower (Figure 6H,I). Collectively, these findings indicate that the mRNA‐based combination therapy induces potent antitumor effects with a favorable safety profile both in the short and long term, supporting its potential for clinical translation.

## 3 Discussion

Previous publications showed that CD47 inhibitors exhibited impressive therapeutic efficacy in pre‐clinical tumor models by promoting macrophage activation and enhancing tumor antigen presentation.^[^ ^32^ ^]^ However, their clinical application was hindered by severe toxic side effects, such as anemia, hemagglutination, fever, and headache, limiting their broader use.^[^ ^12^, ^33^ ^]^ These adverse effects underscored the need for safer and more effective delivery strategies. LNP, which has been widely used in mRNA vaccines, protein replacement therapies, and CRISPR‐based gene editing, provides an effective platform for localized delivery of RNA therapeutics.^[^ ^21^, ^34^ ^]^ In this study, we developed an mRNA‐based CD47 inhibitor formulated in LNP for localized administration. This approach successfully activated macrophages in vitro, and significantly suppressed the growth of immunologically “hot” tumor while minimizing toxic side effects. Nonetheless, consistent with the modest clinical performance of CD47 inhibitors,^[^ ^16^ ^]^ our data also showed that the antitumor efficacy of mRNA‐encoded CD47 inhibitor was limited in immunologically “cold” tumor models. To enhance therapeutic efficacy, current investigations are exploring combination strategies with other immunotherapeutic agents. Previous studies revealed the potential of simultaneously stimulating both the innate and adaptive immune systems to achieve more effective cancer treatment outcomes by integrating CD47 inhibitor with PD‐1/PD‐L1 inhibitors or CAR‐T therapy.^[^ ^17^ ^]^ However, recent phase II clinical trials conducted by ALX Oncology reported that CD47 inhibitor Evorpacept failed to enhance the efficacy of the PD‐1 inhibitor pembrolizumab in patients with advanced HNSCC (NCT04675294, NCT04675333).

One possible explanation for the limited synergy between CD47 and PD‐1 blockade is that PD‐1 inhibitors primarily act on exhausted T cells within the TME, whereas the T cells activated by CD47 inhibition are less responsive to PD‐1 blockade. Given the central role of cytokines in regulating T cell‐mediated immune responses— particularly IL‐12, IL‐15, and IL‐21, which are critical for T cell activation and expansion^[^ ^19^, ^20^ ^]^—we investigated the efficacy of the combination of CD47 inhibitor mRNA and this cytokine cocktail mRNA in cancer treatment. Our results demonstrated that this combination therapy elicited synergistic antitumor effects in both “hot” and “cold” tumors by promoting cDC1 differentiation and systemic activation of cytotoxic T cells, all while maintaining a favorable safety profile. Importantly, the therapy also induced durable antitumor immune memory. Furthermore, lung‐targeted delivery of the mRNA combination using DOTAP‐modified LNP led to robust suppression of lung‐metastatic tumors, offering a promising approach for lung‐specific cancer immunotherapy. These findings underscore the synergistic potential of combining CD47 inhibition with cytokine‐based immune modulation to overcome the immunosuppressive TME. Notably, comparative analysis revealed that this mRNA‐based combination therapy outperformed the combination of CD47 inhibitor mRNA and anti‐PD‐1 antibody. Hematological assessments and inflammatory cytokines profiling indicated minimal risks of anemia or CRS both in the short‐ and long‐term settings. Therefore, our strategy of localized delivery reduces systemic exposure to CD47 inhibitors and cytokines, thereby minimizing off‐target toxicity, while their combined administration produces potent, synergistic antitumor effects—supporting the translational potential of this approach for clinical application.

Tumor metastasis remains the primary cause of cancer‐related mortality.^[^ ^30^, ^35^ ^]^ Despite extensive research focusing on early detection, prevention, and targeted treatment strategies, metastasis continues to present a significant challenge in cancer therapy. In this study, we demonstrated that localized treatment of primary tumors significantly suppressed the progression of lung metastases. These results suggest that the combination of CD47 inhibitor and the cytokine cocktail not only induced a robust systemic T cell‐mediated antitumor response but also established durable immune memory capable of controlling metastatic tumors. This highlights the potential of multi‐modal strategies that simultaneously engage both innate and adaptive immunity to achieve sustained tumor control, offering promising prospects for metastasis immunotherapy. Given the physiological differences between murine and human immune systems, further validation using humanized mouse models and comprehensive toxicological evaluation will be essential before clinical translation. While this cytokine cocktail proved effective, it remains unclear which interleukin(s) play a dominant role in driving the observed synergy. Additionally, other cytokines, such as IL‐2, IL‐7, IFN‐α, IFN‐γ, IL‐4, and IL‐10, are known to enhance T cell‐mediated antitumor immunity.^[^ ^20^, ^36^ ^]^ Future studies should explore whether alternative or optimized cytokine combinations could further improve therapeutic outcomes when used alongside CD47 inhibition.

In mRNA therapeutics, one of the major challenges is the low endosomal escape efficiency of mRNA delivered by LNPs. Previous studies reported that <2% of mRNA molecules delivered via LNPs escape endosomes after cellular uptake.^[^ ^37^ ^]^ While this limited efficiency is often sufficient for vaccines—where small amounts of antigen expression can elicit robust immune responses^[^ ^38^ ^]^— therapeutic proteins typically require higher expression levels to achieve efficacy. Circular mRNA or self‐amplifying mRNA, which produced higher protein yields even at low doses, may offer alternatives for protein replacement therapies.^[^ ^39^ ^]^ Another limitation of current LNP formulations is the immunogenicity associated with PEG. PEG‐specific antibodies, which can be induced by PEG‐lipid components in LNPs, may accelerate the clearance of PEGylated nanoparticles and reduce therapeutic efficacy.^[^ ^40^ ^]^ Various PEG alternatives have been explored to mitigate this issue and extend LNP circulation time.^[^ ^41^ ^]^ Additionally, the ionizable lipid components in LNP have been shown to possess intrinsic adjuvant activity, which contributes to the strong immunogenicity observed in mRNA vaccines.^[^ ^42^ ^]^ While beneficial in vaccine contexts, this adjuvant effect may be undesirable in mRNA‐based protein replacement or long‐term therapies, as it could induce neutralizing antibodies against therapeutic proteins and diminish efficacy. Therefore, future improvements in LNP design should focus on enhancing endosomal escape, reducing immunogenicity, and supporting high‐yield protein expression for diverse therapeutic applications.

## 4 Experimental Section

### Cell Culture

Human and mouse cancer cell lines, including A549, HepG2, THP‐1, 4T1, B16F10, LLC1 and RAW264.7, were obtained from the American Type Culture Collection (ATCC) and maintained in the laboratory. HEK 293T cells were purchased from Thermo Fisher Scientific. All cell lines were cultured in either Dulbecco's Modified Eagle Medium (DMEM) or Roswell Park Memorial Institute (RPMI) 1640 medium, supplemented with 10% fetal bovine serum (FBS) and 1% penicillin/streptomycin. Cells were maintained at 37 °C in a humidified incubator with 5% CO_2_.

### Animal Experiments

All animal experiments were conducted in accordance with protocols approved by the Laboratory Animal Ethics Committee of Southern University of Science and Technology (SUSTech‐JY202308050). All mice used in this study were purchased from GemPharmatech Co., Ltd. and housed under specific pathogen‐free (SPF) conditions. To establish mouse lung‐metastatic tumor models, 2 × 10^5^ of cancer cells were injected intravenously into each mouse. For mouse subcutaneous tumor models, 5 × 10^5^ of cancer cells were injected subcutaneously in the right flank of mice. Mouse strains NOD SCID, C57BL/6, and BALB/c were selected based on the cancer cell lines used for injection. One week after tumor establishment, the mice were randomly assigned to different groups and received either 20 µg of mRNA intravenously or 5–8 µg intratumorally per dose. A total of three to four treatments were administered. The anti‐PD‐1 antibody (Leinco, clone RMP1‐14) was administered intraperitoneally at a dose of 10 mg kg^−1^ per week. Traditional LNP was used for the treatment of subcutaneous tumors, while lung‐targeted LNP was employed for lung‐metastatic tumors. Tumor tissues were harvested at the end of the experiments for subsequent analysis.

For the tumor rechallenge experiment, mice previously treated and cured were reinoculated with 5 × 10^6^ LLC1 cells four months after surgical removal of the primary tumors. For blood test, peripheral blood samples were collected from the mice and evaluated using heamatology analyzer (DYMIND DF52 Vet). For bioluminescent imaging assay, mice were treated intravenously with 5 µg of luciferase mRNA. Six hours post‐treatment, fluorescein sodium salt was injected intraperitoneally, followed by bioluminescent imaging.

### Plasmids and In Vitro Transcription

The coding sequences of A4‐IgG1 and CV1‐IgG1 were synthesized by GenScript and cloned into the pVAX1 vector between the BamHI and NotI restriction sites. The genes encoding mouse IL‐12 (IL12a, NM_001159424.3; IL12b, NM_001303244.1), IL‐15 (NM_008357.3), and IL‐21 (NM_001291041.1) were also cloned into a single pVAX1 vector, linked via P2A sequences. A 5′ untranslated region (UTR) from human herpesvirus and a 3′ UTR from human alpha‐1‐globulin were incorporated at the respective ends of the open reading frame to enhance expression.

For in vitro transcription, the plasmids were linearized using either XbaI or XhoI restriction enzymes and purified with a Phenol:Chloroform:Isoamyl Alcohol solution (25:24:1). The linearized DNA served as the template for mRNA synthesis, during which uridine was substituted with N1‐methylpseudouridine to improve stability and reduce immunogenicity. Transcription reactions were carried out at 37 °C for 2 h following the manufacturer's instructions. The resulting mRNAs were purified using a 2.5 M LiCl solution and stored at −80 °C until use.

### mRNA Encapsulation

Lipids used in the formulation of traditional LNPs (DLin‐MC3, cholesterol, DSPC, and DMG‐PEG2000 at a molar ratio of 50:38.5:10:1.5) or lung‐targeted LNPs (DLin‐MC3, cholesterol, DSPC, DMG‐PEG2000, and DOTAP at a molar ratio of 25:19.25:5:0.75:50) were dissolved in ethanol. mRNA was diluted in 50 mm sodium citrate buffer (pH 4.0). The ethanol‐based lipid solution was rapidly mixed with the aqueous mRNA solution using a microfluidic device at a flow rate ratio of 1:3 (ethanol to aqueous), with an N/P ratio of 6 and an mRNA‐to‐total lipid weight ratio of 20:1. Following formulation, the solvent was exchanged with PBS via ultrafiltration.

The encapsulation efficiency and concentration of the resulting mRNA‐loaded nanoparticles were assessed using the RiboGreen RNA Assay Kit. Particle size and polydispersity index (PdI) were characterized by dynamic light scattering using a Zetasizer Nano ZS. The formulated nanoparticles were stored at 4 °C and used within three days of preparation.

### Phagocytosis Assay In Vitro

To obtain primary macrophages of mice, bone marrow cells were isolated from BALB/c mice and cultured in RPMI 1640 medium with 10% FBS, 1% penicillin/streptomycin, and 10 ng ml^−1^ murine M‐CSF (Peprotech) at 37 °C for 10 days. 4T1 cells were labeled with CFSE dye (Invitrogen) and co‐cultured with either primary macrophages at a 10:1 effector‐to‐target (E:T) ratio or with Raw264.7 cells at a 5:1 E:T ratio for 2 h. The macrophages that engulfed 4T1 cells exhibited green fluorescence. These green macrophages were analyzed by fluorescence microscope or flow cytometry.

### Cell Lysis and Western Blot

The cells were harvested after digestion with trypsin and washed by PBS, followed by lysing with RIPA lysis buffer containing 1% protease inhibitor cocktail and incubating on ice for 30 min. The supernatant from cell lysate was collected after centrifugation (12 000 rpm) at 4 °C for 10 min. For tissue lysis, the samples were cut into small pieces and homogenized by tissue grinder in RIPA lysis buffer. The tissue lysates were incubated on ice for 30 min and centrifuged at 12,000 rpm and 4 °C for 10 min. The total protein concentration of all lysates was determined with BCA assay kit (Thermo Fisher Scientific).

For western blot analysis, 30 µg of total protein from each sample was loaded into PAGE gel and transferred to a PVDF membrane (Merk) using the Wet Protein Transfer System (eBlot L1, GenScript). After blocking non‐specific binding with 5% defatted milk (Sangon, #A600669) at room temperature for 1 h, the membranes were incubated with antibodies at 4 °C. Immunoblot signal was determined with an imaging system. The antibodies used in this study include: anti‐tubulin (proteintech, HRP‐66031), anti‐GAPDH (proteintech, HRP‐60004), anti‐human IgG FC (abcam, #97225).

### Real‐Time PCR

The total RNA of the tumor tissues were extracted by TRIzol Reagent (Life Technologies) according to the manufacturer's instruction. Briefly, the fresh tumor tissues were cut into small pieces, and 1 ml of TRIzol Reagent was added, followed by thorough mixing. The mixture was incubated at room temperature for 5 min, after which 200 µl of chloroform was added. Following a 3‐min incubation at room temperature, the mixture was centrifuged at 12 000 rpm and 4 °C for 15 min. The supernatants were collected and mixed with 500 µl of isopropanol. After a 10‐min incubation, the RNA was pelleted by centrifugation at 12 000 rpm and 4 °C for 10 min, washed with 75% ethanol, and dissolved in RNase‐free water. The total RNA was stored at −80 °C until further use.

The complementary DNA (cDNA) generation was performed with SYBR qPCR Master Mix (Vazyme) following the manufacturer's protocol. The detection agent III RT SuperMix (Vazyme) was used for real‐time PCR determination according to the manufacture's instruction. The primers used in real‐time PCR were listed in Table S1 (Supporting Information).

### Analysis of Tumor‐Infiltrating Immune Cells

Tumor tissues were cut into small pieces and digested with collagenase (Sigma, #C9891), hyaluronidase (Sigma, #H3506), and DNase I (Roche, #10104159001) at 37 °C for 30 min. The digestion was stopped by adding an equal volume of medium containing 10% FBS. The resulting cell suspension was filtered through a 70 µm cell strainer, and the single cells were washed twice with PBS. The cell pellets were resuspended with ACK Lysing Buffer (0.15 m NH4Cl, 10 mm KHCO3, 0.1 mm EDTA, pH 7.2) and incubated at room temperature within 5 min to remove red blood cells. The isolated cells were stained with LIVE/DEAD dye (Invitrogen, #L34965) in PBS, followed by FcR blocking (BD, #553141) and antibodies staining in FACS Buffer (2 mm EDTA, 2% FBS in PBS) at 4 °C for 30 min. For intracellular cytokine staining, the cells were fixed and permeabilized using Cytofix/Cytoperm Fixation/Permeabilization Kit (BD Bioscience, #554714). The antibodies used for flow cytometry analysis include: anti‐human CD47 (BioLegend, #323123), anti‐human IgG FC (BioLegend, #410707), anti‐mouse CD47 (BioLegend, #127513), anti‐mouse CD45 (BioLegend, #147714), anti‐mouse CD3 (BioLegend, #100203), anti‐mouse CD8 (BioLegend, #100712), anti‐mouse CD4 (BioLegend, #100408), anti‐mouse CD11b (BioLegend, #101235), anti‐mouse CD80 (BioLegend, #104713), anti‐mouse CD86 (BioLegend, #105005), anti‐mouse CD163 (BioLegend, #155307), anti‐mouse CD49b (BioLegend, #108909), anti‐mouse F4/80 (BioLegend, #123107), anti‐mouse IFN‐γ (BioLegend, #505829), anti‐mouse CD11c (BioLegend, #117329), anti‐mouse CD27 (BioLegend, #124209), anti‐mouse I‐A/I‐E (BioLegend, #107607), anti‐mouse Granzyme B (BioLegend, #515410).

### Hematoxylin and Eosin Staining

The tumor tissues were embedded with paraffin after fixation in formalin. The paraffin‐embedded tissue sections were stained with hematoxylin and eosin using the HistoCore Arcadia Embedding System. The slides were scanned and analyzed using the Aperio CS2 digital pathology system.

### Purification of A4‐IgG1 Fusion Protein

The plasmid was transfected into 293T cells using PEI transfection reagent, and the supernatant was collected 48 h post‐transfection. The A4‐IgG1 fusion protein in the supernatant was purified using protein A resin (GenScript). Briefly, the supernatant was diluted 1:1 with Binding/Wash Buffer (0.15 m NaCl, 20 mm Na_2_HPO_4_, pH 7.0) and kept on ice. The protein A resin was fully resuspended, and 2 mL of resin was transferred to a new column. The column was equilibrated with 1 mL of Binding/Wash Buffer, followed by an additional 5 mL of Binding/Wash Buffer at a flow rate of 1 mL min^−1^. The diluted supernatant was then loaded onto the column and allowed to flow through by gravity at a flow rate of ≈1 mL min^−1^. The column was washed with 30 mL of Binding/Wash Buffer at a flow rate of ≈2 mL min^−1^. The A4‐IgG1 fusion protein was eluted using Elution Buffer (0.1 m glycine, pH 3.0), with a total elution volume of 10–15 mL at a flow rate of 1 mL min^−1^. The eluate was immediately neutralized to pH 7.4 by adding Neutralization Buffer (1 m Tris‐HCl, pH 8.5) at 1/10th the volume of the total eluate. The solvent for the A4‐IgG1 fusion protein was replaced with PBS via ultrafiltration, and the protein concentration was determined using the BCA assay kit (Thermo Fisher Scientific).

### RNA Sequencing

To perform RNA sequencing analysis of T cells, spleens were cut into small pieces and placed in a cell strainer, followed by grinding with a syringe plunger to obtain single‐cell suspensions. The single cells were stained with anti‐CD45 and anti‐CD3 antibodies, and CD45^+^ CD3^+^ T cells were sorted using flow cytometry. Propidium iodide (PI) was used to distinguish live and dead cells. Total RNA was extracted from the sorted CD45^+^ CD3^+^ T cells with TRIzol Reagent (Life Technologies) according to the manufacturer's instruction. RNA sequencing was performed by Novogene Co., Ltd. using the DNBSEQ‐T7 platform. Gene set enrichment analysis (GSEA) was performed according to the previously described protocol.^[^ ^44^ ^]^

### ELISA Assay

Peripheral blood samples were collected into anticoagulant tubes and centrifuged at 2000 × g for 10 min at 4 °C. The serum from the upper layer was carefully harvested and stored at −80 °C until further use. The concentrations of cytokines IL‐6 and TNF‐α were measured using Mouse Precoated ELISA Kits (Dakewe Biotech, #1210602 for IL‐6 and #1217202 for TNF‐α) according to the manufacturer's instructions. Briefly, 100 µl of appropriately diluted serum samples was added to the precoated wells, followed by the addition of 50 µl of 1× Biotinylated Antibody to each well. After incubation at 37 °C for 90 min, the wells were washed at least four times with the provided Washing Buffer. Subsequently, 50 µl of Streptavidin‐HRP was added to each well and incubated at 37 °C for 30 min. Following another wash step, 100 µl of TMB Substrate was added and incubated for 20 min at 37 °C. The reaction was then terminated by adding 100 µl of Stop Solution to each well. Absorbance was measured at 450 nm with a reference wavelength of 630 nm within 10 min. The final OD value for each sample was calculated as OD450 nm minus OD630 nm.

### Statistical Analysis

The statistical significance was analyzed using an unpaired two‐tailed Student's t‐test or one‐way ANOVA with Tukey's multiple comparisons tests unless otherwise specified in the figure legends. All statistical analyses were performed in GraphPad Prism. *p < 0.05, **p < 0.01, ***p < 0.001, and ****p < 0.0001, Mean ± SD, ns means non‐significant.

## Conflict of Interest

The authors declare no conflict of interest.

## Supporting information



Supporting Information

## Data Availability

The data that support the findings of this study are available from the corresponding author upon reasonable request.

## References

[1] N. M. Anderson , M. C. Simon , Curr. Biol. 2020, 30, R921. 32810447 10.1016/j.cub.2020.06.081 PMC8194051

[2] M. Binnewies , E. W. Roberts , K. Kersten , V. Chan , D. F. Fearon , M. Merad , L. M. Coussens , D. I. Gabrilovich , S. Ostrand‐Rosenberg , C. C. Hedrick , R. H. Vonderheide , M. J. Pittet , R. K. Jain , W. Zou , T. K. Howcroft , E. C. Woodhouse , R. A. Weinberg , M. F. Krummel , Nat. Med. 2018, 24, 541. 29686425 10.1038/s41591-018-0014-x PMC5998822

[3] a) X. Lei , Y. Lei , J. K. Li , W. X. Du , R. G. Li , J. Yang , J. Li , F. Li , H. B. Tan , Cancer Lett. 2020, 470, 126; 31730903 10.1016/j.canlet.2019.11.009

[4] a) X. He , C. Xu , Cell Res. 2020, 30, 660; 32467592 10.1038/s41422-020-0343-4 PMC7395714

[5] C. Robert , Nat. Commun. 2020, 11, 3801. 32732879 10.1038/s41467-020-17670-y PMC7393098

[6] R. C. Sterner , R. M. Sterner , Blood Cancer J. 2021, 11, 69. 33824268 10.1038/s41408-021-00459-7 PMC8024391

[7] M. E. W. Logtenberg , F. A. Scheeren , T. N. Schumacher , Immunity 2020, 52, 742. 32433947 10.1016/j.immuni.2020.04.011 PMC7340539

[8] a) E. J. Brown , W. A. Frazier , Trends Cell Biol. 2001, 11, 130; 11306274 10.1016/s0962-8924(00)01906-1

[9] P. A. Oldenborg , A. Zheleznyak , Y. F. Fang , C. F. Lagenaur , H. D. Gresham , F. P. Lindberg , Science 2000, 288, 2051. 10856220 10.1126/science.288.5473.2051

[10] B. R. Blazar , F. P. Lindberg , E. Ingulli , A. Panoskaltsis‐Mortari , P. A. Oldenborg , K. Iizuka , W. M. Yokoyama , P. A. Taylor , J. Exp. Med. 2001, 194, 541. 11514609 10.1084/jem.194.4.541 PMC2193501

[11] M. P. Chao , I. L. Weissman , R. Majeti , Curr. Opin. Immunol. 2012, 24, 225. 22310103 10.1016/j.coi.2012.01.010 PMC3319521

[12] Z. Jiang , H. Sun , J. Yu , W. Tian , Y. Song , J. Hematol. Oncol. 2021, 14, 180. 34717705 10.1186/s13045-021-01197-w PMC8557524

[13] a) S. B. Willingham , J. P. Volkmer , A. J. Gentles , D. Sahoo , P. Dalerba , S. S. Mitra , J. Wang , H. Contreras‐Trujillo , R. Martin , J. D. Cohen , P. Lovelace , F. A. Scheeren , M. P. Chao , K. Weiskopf , C. Tang , A. K. Volkmer , T. J. Naik , T. A. Storm , A. R. Mosley , B. Edris , S. M. Schmid , C. K. Sun , M. S. Chua , O. Murillo , P. Rajendran , A. C. Cha , R. K. Chin , D. Kim , M. Adorno , T. Raveh , et al., Proc. Natl. Acad. Sci. USA 2012, 109, 6662; 22451913 10.1073/pnas.1121623109 PMC3340046

[14] a) X. Liu , Y. Pu , K. Cron , L. Deng , J. Kline , W. A. Frazier , H. Xu , H. Peng , Y. X. Fu , M. M. Xu , Nat. Med. 2015, 21, 1209; 26322579 10.1038/nm.3931 PMC4598283

[15] J. Son , R. C. Hsieh , H. Y. Lin , K. J. Krause , Y. Yuan , A. B. Biter , J. Welsh , M. A. Curran , D. S. Hong , Front. Immunol. 2022, 13, 1027235. 36439116 10.3389/fimmu.2022.1027235 PMC9691650

[16] R. Bouwstra , T. van Meerten , E. Bremer , Clin. Transl. Med. 2022, 12, 943. 10.1002/ctm2.943 PMC9339239 35908284

[17] a) M. M. Dacek , K. G. Kurtz , P. Wallisch , S. A. Pierre , S. Khayat , C. M. Bourne , T. J. Gardner , K. C. Vogt , N. Aquino , A. Younes , D. A. Scheinberg , Blood 2023, 141, 2003; 36696633 10.1182/blood.2022016101 PMC10163312

[18] a) J. M. Curtsinger , M. F. Mescher , Curr. Opin. Immunol. 2010, 22, 333; 20363604 10.1016/j.coi.2010.02.013 PMC2891062

[19] a) K. C. Conlon , M. D. Miljkovic , T. A. Waldmann , J. Interf. Cytok. Res. 2019, 39, 6; 10.1089/jir.2018.0019 PMC6350412 29889594

[20] a) D. J. Propper , F. R. Balkwill , Nat. Rev. Clin. Oncol. 2022, 19, 237; 34997230 10.1038/s41571-021-00588-9

[21] X. C. Hou , T. Zaks , R. Langer , Y. Z. Dong , Nat. Rev. Mater. 2021, 6, 1078. 34394960 10.1038/s41578-021-00358-0 PMC8353930

[22] N. Chaudhary , D. Weissman , K. A. Whitehead , Nat. Rev. Drug Discovery 2021, 20, 817. 34433919 10.1038/s41573-021-00283-5 PMC8386155

[23] a) T. Vavilis , E. Stamoula , A. Ainatzoglou , A. Sachinidis , M. Lamprinou , I. Dardalas , I. S. Vizirianakis , Pharmaceutics 2023, 15, 166; 36678793 10.3390/pharmaceutics15010166 PMC9866414

[24] a) J. Popovitz , R. Sharma , R. Hoshyar , B. Soo Kim , N. Murthy , K. Lee , Adv. Drug Delivery Rev. 2023, 200, 115026; 10.1016/j.addr.2023.115026 37516409

[25] Q. Cheng , T. Wei , L. Farbiak , L. T. Johnson , S. A. Dilliard , D. J. Siegwart , Nat. Nanotechnol. 2020, 15, 313. 32251383 10.1038/s41565-020-0669-6 PMC7735425

[26] Y. Sun , S. Chatterjee , X. Lian , Z. Traylor , S. R. Sattiraju , Y. Xiao , S. A. Dilliard , Y. C. Sung , M. Kim , S. M. Lee , S. Moore , X. Wang , D. Zhang , S. Wu , P. Basak , J. Wang , J. Liu , R. J. Mann , D. F. LePage , W. Jiang , S. Abid , M. Hennig , A. Martinez , B. A. Wustman , D. J. Lockhart , R. Jain , R. A. Conlon , M. L. Drumm , C. A. Hodges , D. J. Siegwart , Science 2024, 384, 1196. 38870301 10.1126/science.adk9428 PMC12208706

[27] J. T. Sockolosky , M. Dougan , J. R. Ingram , C. C. Ho , M. J. Kauke , S. C. Almo , H. L. Ploegh , K. C. Garcia , Proc. Natl. Acad. Sci. USA 2016, 113, E2646. 27091975 10.1073/pnas.1604268113 PMC4868409

[28] J. M. Caaveiro , M. Kiyoshi , K. Tsumoto , Immunol. Rev. 2015, 268, 201. 26497522 10.1111/imr.12365

[29] M. M. Xu , Y. Pu , D. Han , Y. Shi , X. Cao , H. Liang , X. Chen , X. D. Li , L. Deng , Z. J. Chen , R. R. Weichselbaum , Y. X. Fu , Immunity 2017, 47, 363. 28801234 10.1016/j.immuni.2017.07.016 PMC5564225

[30] X. Guan , Acta Pharm. Sin. B 2015, 5, 402. 26579471 10.1016/j.apsb.2015.07.005 PMC4629446

[31] J. Witten , Y. Hu , R. Langer , D. G. Anderson , Proc. Natl. Acad. Sci. USA 2024, 121, 2307798120. 10.1073/pnas.2307798120 PMC10945842 38437569

[32] a) E. R. Unanue , Proc. Natl. Acad. Sci. USA 2013, 110, 10886; 23784781 10.1073/pnas.1308463110 PMC3704033

[33] R. Maute , J. Xu , I. L. Weissman , Immunooncol. Technol. 2022, 13, 100070. 35754851 10.1016/j.iotech.2022.100070 PMC9216458

[34] R. Tenchov , R. Bird , A. E. Curtze , Q. Q. Zhou , ACS Nano 2021, 15, 16982. 34181394 10.1021/acsnano.1c04996

[35] T. N. Seyfried , L. C. Huysentruyt , Crit. Rev. Oncog. 2013, 18, 43. 23237552 10.1615/critrevoncog.v18.i1-2.40 PMC3597235

[36] a) B. Feng , Z. Bai , X. Zhou , Y. Zhao , Y. Q. Xie , X. Huang , Y. Liu , T. Enbar , R. Li , Y. Wang , M. Gao , L. Bonati , M. W. Peng , W. Li , B. Tao , M. Charmoy , W. Held , J. J. Melenhorst , R. Fan , Y. Guo , L. Tang , Nature 2024, 634, 712; 39322665 10.1038/s41586-024-07962-4 PMC11485240

[37] a) S. Chatterjee , E. Kon , P. Sharma , D. Peer , Proc. Natl. Acad. Sci. USA 2024, 121, 2307800120; 10.1073/pnas.2307800120 PMC10945858 38437552

[38] N. Pardi , M. J. Hogan , F. W. Porter , D. Weissman , Nat. Rev. Drug Discovery 2018, 17, 261. 29326426 10.1038/nrd.2017.243 PMC5906799

[39] a) R. Chen , S. K. Wang , J. A. Belk , L. Amaya , Z. Li , A. Cardenas , B. T. Abe , C. K. Chen , P. A. Wender , H. Y. Chang , Nat. Biotechnol. 2023, 41, 262; 35851375 10.1038/s41587-022-01393-0 PMC9931579

[40] a) Y. Ju , J. M. Carreno , V. Simon , K. Dawson , F. Krammer , S. J. Kent , Nat. Rev. Immunol. 2023, 23, 135; 36539526 10.1038/s41577-022-00825-x PMC9764299

[41] a) A. Sanchez , D. Loughrey , E. S. Echeverri , S. G. Huayamares , A. Radmand , K. Paunovska , M. Hatit , K. E. Tiegreen , P. J. Santangelo , J. E. Dahlman , Adv. Healthcare Mater. 2024, 13, 2304033; 10.1002/adhm.202304033 38318754

[42] M. G. Alameh , I. Tombacz , E. Bettini , K. Lederer , C. Sittplangkoon , J. R. Wilmore , B. T. Gaudette , O. Y. Soliman , M. Pine , P. Hicks , T. B. Manzoni , J. J. Knox , J. L. Johnson , D. Laczko , H. Muramatsu , B. Davis , W. Meng , A. M. Rosenfeld , S. Strohmeier , P. J. C. Lin , B. L. Mui , Y. K. Tam , K. Kariko , A. Jacquet , F. Krammer , P. Bates , M. P. Cancro , D. Weissman , E. T. Luning Prak , D. Allman , et al., Immunity 2022, 55, 1136. 35704995 10.1016/j.immuni.2022.05.007 PMC9195404

[43] E. Rohner , R. Yang , K. S. Foo , A. Goedel , K. R. Chien , Nat. Biotechnol. 2022, 40, 1586. 36329321 10.1038/s41587-022-01491-z

[44] A. Subramanian , P. Tamayo , V. K. Mootha , S. Mukherjee , B. L. Ebert , M. A. Gillette , A. Paulovich , S. L. Pomeroy , T. R. Golub , E. S. Lander , J. P. Mesirov , Proc. Natl. Acad. Sci. USA 2005, 102, 15545. 16199517 10.1073/pnas.0506580102 PMC1239896

