# Supplementary material for: Localized Delivery of the mRNAs Encoding CD47 Inhibitor and Interleukins 12, 15, and 21 Elicits Robust Antitumor Immunity
Source: Adv Sci (Weinh). 2025 Jul 13;12(37):e17205. doi: 10.1002/advs.202417205 (PMC12499455; doi:10.1002/advs.202417205)
Supplement: Supplementary file 1 — Supporting Information [file ADVS-12-e17205-s001.docx]

Supporting Information

Localized Delivery of the mRNAs Encoding CD47 Inhibitor and Interleukins 12, 15, and 21 Elicits Robust Antitumor Immunity

Tao Jiang*, Shuaiyang Jing, Haojun Li, Bao Xiao, Jiahui Jin, Xiu Sun, Juan Wang, Jing Liang, Tongze Cai, Huili Hu, Meilan Wei, Xuanrui Zhong, Yang Ji*, Peng George Wang* and Jianlong Zhou*

**Supplementary figures and tables**


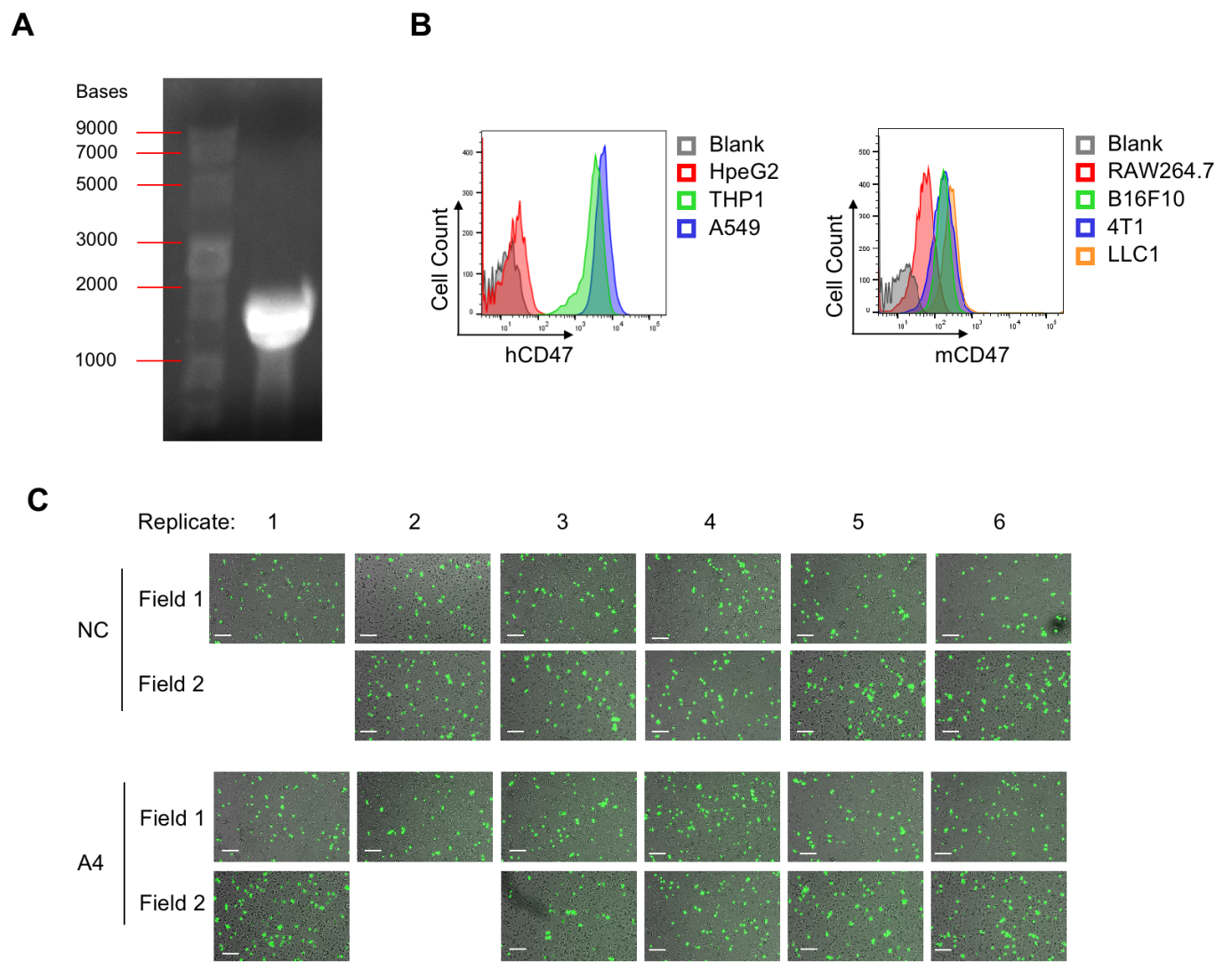


Figure S1. mRNA-encoded CD47 inhibitor activated macrophage phagocytosis in vitro. A) The electrophoresis gel image of A4 mRNA produced from in vitro transcription. B) Flow cytometry analysis of CD47 expression level in human cancer cell lines (HepG2, THP1 and A549) and mouse cancer cell lines (RAW264.7, 4T1 and B16F10). C) The images from all replicate experiments of primary macrophages phagocytosis. Two different imaging fields were presented for each replicate. 4T1 cancer cells were labeled by CFSE dye. Scale bar: 300 μm.


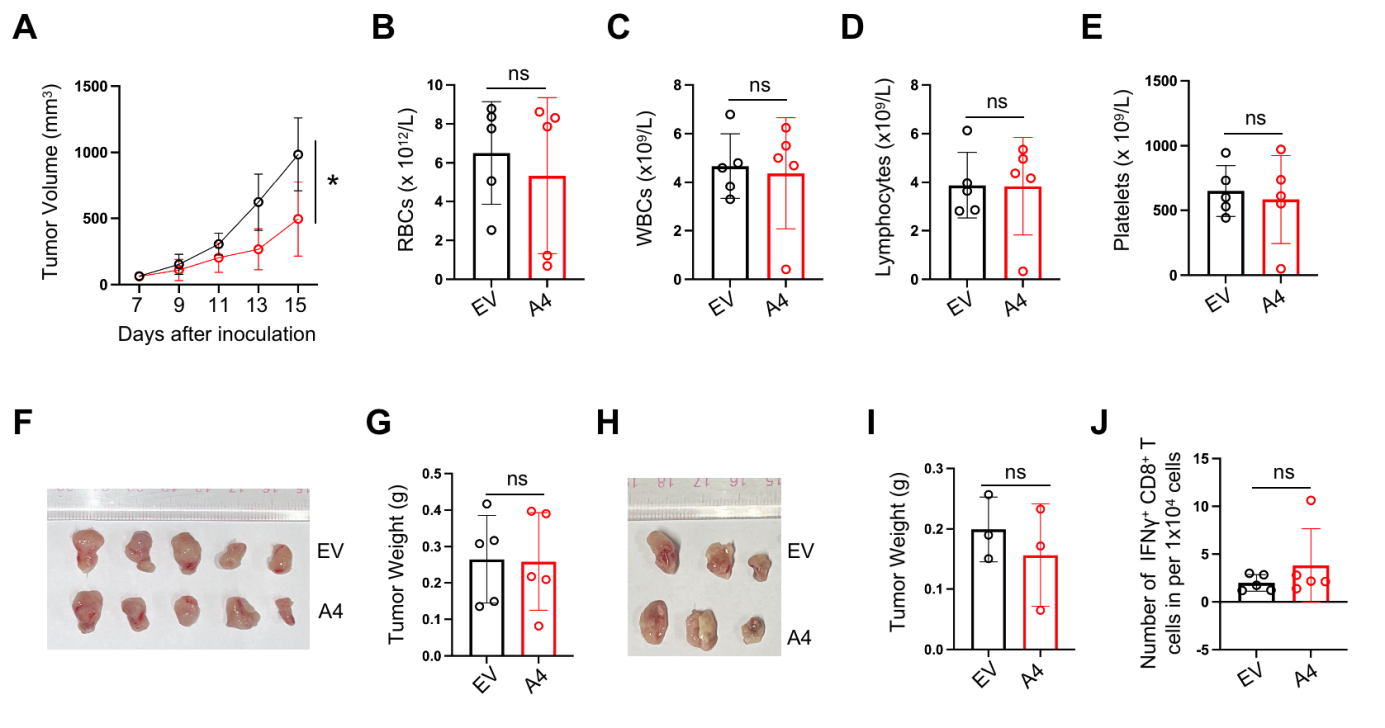


Figure S2. mRNA-encoded CD47 Inhibitor Promoted cDC1 Differentiation. A) The tumor volume of melanoma was plotted. n=5. B-E) Blood test of the tumor-bearing mice following A4 mRNA treatment. The numbers of red blood cells (B), white blood cells (C), lymphocytes (D), platetlets (E) were quantified. n=5. F,G) The images of 4T1 tumors were showed (F) and the tumor weight was quantified (G) after treatments. n=5. H,I) The images of LLC1 tumors were showed (H) and the tumor weight was quantified (I) after treatments. n=3. J)Bar chart quantifying the number of infiltrating IFN-γ^+^ CD8^+^ T cells in tumor. n=5. In all panels, significance was determined by unpaired two-tailed t-test. Mean ± SD, ns: not significant.


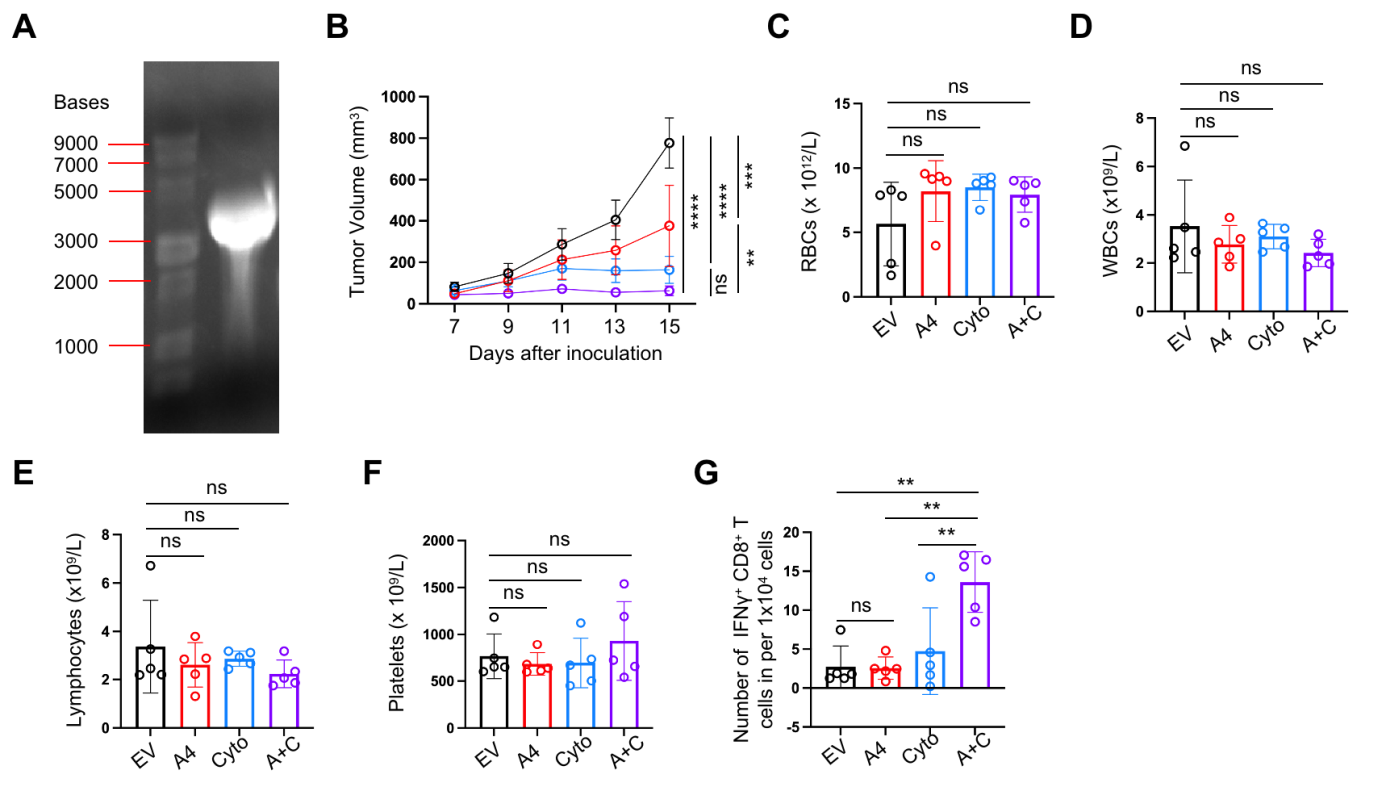


Figure S3. Combination of CD47 inhibitor and a cytokine cocktail synergistically suppressed tumor progression. A) The electrophoresis gel image of A4 mRNA produced from in vitro transcription. B) The tumor volume of melanoma was plotted. n=5. C-F) Blood test of the tumor-bearing mice following mRNA-based treatments. The numbers of red blood cells (C), white blood cells (D), lymphocytes (E), platetlets (F) were quantified. n=5. G) Bar chart quantifying the number of infiltrating IFN-γ^+^ CD8^+^ T cells in tumor. n=5. In all panels, significance was determined by one-way ANOVA with Tukey’s multiple comparisons tests. Mean ± SD, ns: not significant. **P<0.01.


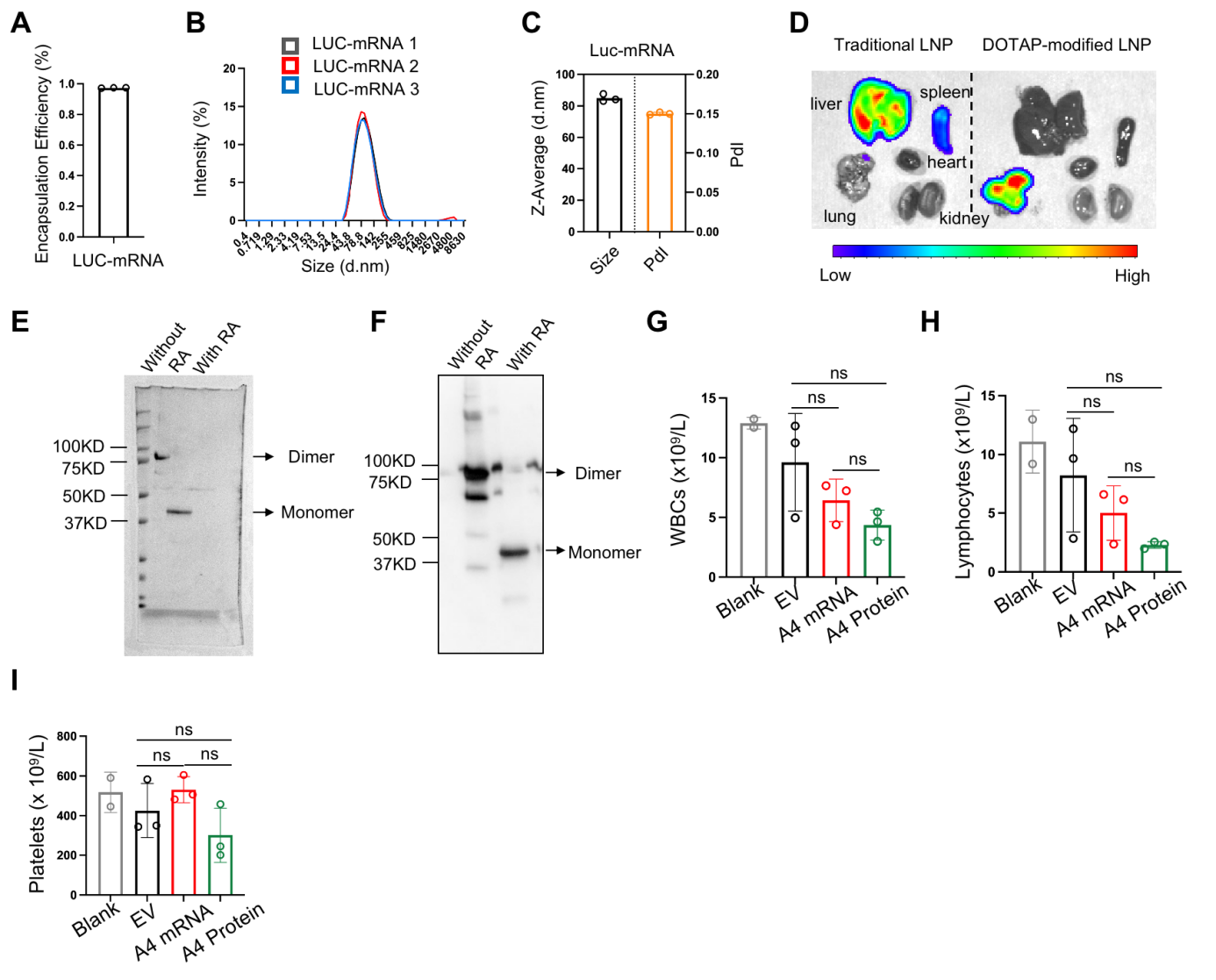


Figure S4. The safety profile of lung-targeted therapy. A-C) The characterization of A4 mRNA encapsulated by DOTAP-modified DLin-MC3 LNP. The encapsulation efficiency (A), particle size (B,C) and polydispersity index (PdI) (C) were showed. n= 3. D) Bioluminescent imaging of major organs from the mice treated intravenously with 5 μg of firefly luciferase mRNA encapsulated in either traditional DLin-MC3 LNPs or DOTAP-modified DLin-MC3 LNPs. Organs were collected for imaging six hours post-treatment. E,F) The coomassie blue staining of purified A4-IgG1 protein in PAGE gel (E) and the western blot analysis of purified A4-IgG1 protein blotting with anti-human IgG antibody (F). RA: reduced agent. G-I) Blood test of the healthy mice following the treatments of A4 mRNA and A4-IgG1 protein. The numbers of white blood cells (G), lymphocytes (H), platetlets (I) were quantified. n=3. In all panels, significance was determined by one-way ANOVA with Tukey’s multiple comparisons tests. Mean ± SD, ns: not significant.


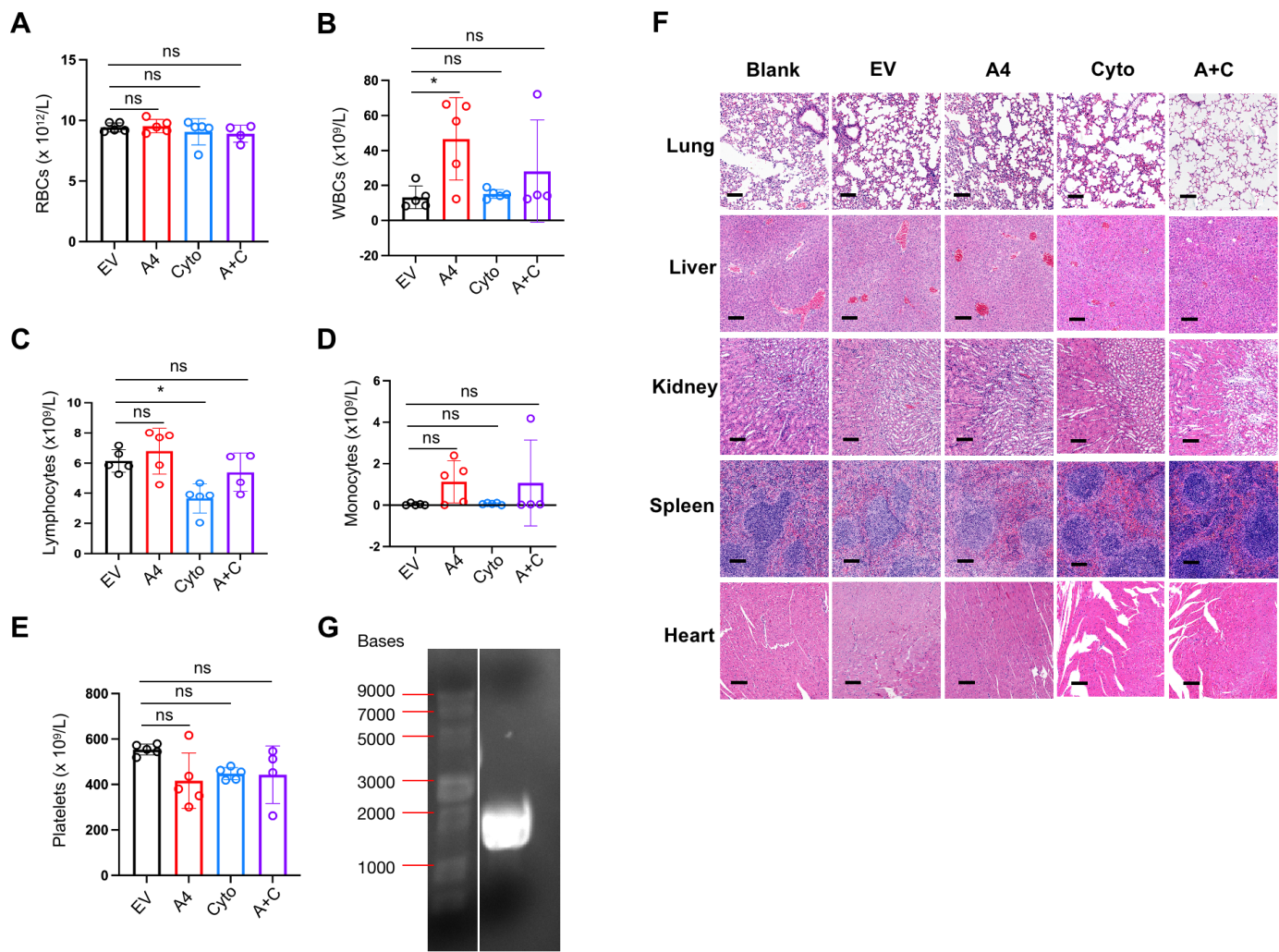


Figure S5. The safety profile of mRNA-based treatments. A-E) Blood test of the tumor-bearing mice following the mRNA-based treatments. The numbers of red blood cells (A), white blood cells (B), lymphocytes (C), monocytes (D), platetlets (E) were quantified. n=4-5. F) The representative images of hematoxylin and eosin staining of lungs, livers, kidneys, spleens and hearts of the healthy mice receiving mRNA-based-treatments. The scale bars represent 125 μm. G) The electrophoresis gel image of CV1 mRNA produced from in vitro transcription. In all panels, significance was determined by one-way ANOVA with Tukey’s multiple comparisons tests. Mean ± SD, ns: not significant. *P<0.05.


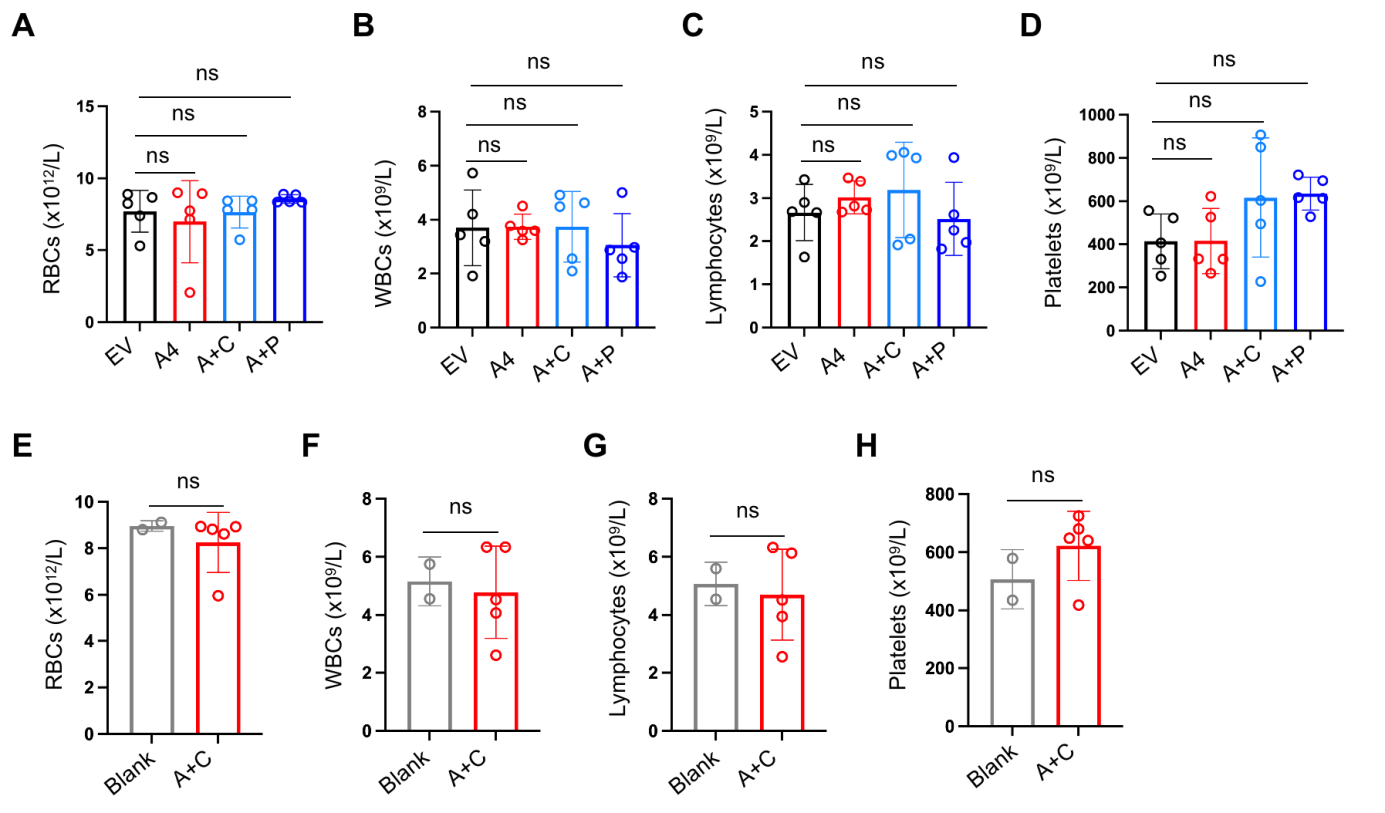


Figure S6. The long-term safety profile of mRNA-based treatments. A-D) Blood test of the tumor-bearing mice following the mRNA-based treatments. The numbers of red blood cells (A), white blood cells (B), lymphocytes (C), and platetlets (D) were quantified. n=5. Significance was determined by one-way ANOVA with Tukey’s multiple comparisons tests. Mean ± SD, ns: not significant. E-H) Blood test of the tumor-free mice that survived for over eight months after tumor rechallenge. The numbers of red blood cells (E), white blood cells (F), lymphocytes (G), and platetlets (H) were quantified. n(blank)=2, n(A+C)=5. Significance was determined by unpaired two-tailed t-test. Mean ± SD, ns: not significant.

Table S1. Table of primers used in real-time PCR.

| Gene | Primer Sequence |
| --- | --- |
| IFN-γ | Forward: CAGCAACAGCAAGGCGAAAAAGG |
|  | Reverse: TTTCCGCTTCCTGAGGCTGGAT |
| IL6 | Forward: TACCACTTCACAAGTCGGAGGC |
|  | Reverse: CTGCAAGTGCATCATCGTTGTTC |
| IL10 | Forward: CGGGAAGACAATAACTGCACCC |
|  | Reverse: CGGTTAGCAGTATGTTGTCCAGC |
| TGF-β | Forward: TGATACGCCTGAGTGGCTGTCT |
|  | Reverse: CACAAGAGCAGTGAGCGCTGAA |
| IL1β | Forward: TGGACCTTCCAGGATGAGGACA |
|  | Reverse: GTTCATCTCGGAGCCTGTAGTG |
| TNF-ɑ | Forward: GGTGCCTATGTCTCAGCCTCTT |
|  | Reverse: GCCATAGAACTGATGAGAGGGAG |
| PD-L1 | Forward: TGCGGACTACAAGCGAATCACG |
|  | Reverse: CTCAGCTTCTGGATAACCCTCG |
| CXCL9 | Forward: CCTAGTGATAAGGAATGCACGATG |
|  | Reverse: CTAGGCAGGTTTGATCTCCGTTC |
| CXCL10 | Forward: ATCATCCCTGCGAGCCTATCCT |
|  | Reverse: GACCTTTTTTGGCTAAACGCTTTC |
| IFN-β | Forward: GCCTTTGCCATCCAAGAGATGC |
|  | Reverse: ACACTGTCTGCTGGTGGAGTTC |
| CCL2 | Forward: GCTACAAGAGGATCACCAGCAG |
|  | Reverse: GTCTGGACCCATTCCTTCTTGG |
| IDO1 | Forward: GCAGACTGTGTCCTGGCAAACT |
|  | Reverse: AGAGACGAGGAAGAAGCCCTTG |
| β-actin | Forward: CATTGCTGACAGGATGCAGAAGG |
|  | Reverse: TGCTGGAAGGTGGACAGTGAGG |
